# Sirtuin 3 protects against anesthesia/surgery-induced cognitive decline in aged mice by suppressing hippocampal neuroinflammation

**DOI:** 10.1186/s12974-021-02089-z

**Published:** 2021-02-04

**Authors:** Qiang Liu, Yi-Man Sun, Hui Huang, Chen Chen, Jie Wan, Lin-Hui Ma, Yin-Ying Sun, Hui-Hui Miao, Yu-Qing Wu

**Affiliations:** 1 Jiangsu Province Key Laboratory of Anesthesiology, Xuzhou Medical University, Tongshan Road 209, Xuzhou, 221004 P.R. China; 2 Department of Anesthesiology, Beijing Friendship Hospital, Capital Medical University, Beijing, 100050 P.R. China

**Keywords:** SIRT3, Postoperative cognitive dysfunction, Mitochondrial oxidative stress, Neuroinflammation, Microglia, Synaptic plasticity

## Abstract

**Background:**

Postoperative cognitive dysfunction (POCD) is a very common complication that might increase the morbidity and mortality of elderly patients after surgery. However, the mechanism of POCD remains largely unknown. The NAD-dependent deacetylase protein Sirtuin 3 (SIRT3) is located in the mitochondria and regulates mitochondrial function. SIRT3 is the only sirtuin that specifically plays a role in extending lifespan in humans and is associated with neurodegenerative diseases. Therefore, the aim of this study was to evaluate the effect of SIRT3 on anesthesia/surgery-induced cognitive impairment in aged mice.

**Methods:**

SIRT3 expression levels were decreased after surgery. For the interventional study, an adeno-associated virus (AAV)-SIRT3 vector or an empty vector was microinjected into hippocampal CA1 region before anesthesia/surgery. Western blotting, immunofluorescence staining, and enzyme-linked immune-sorbent assay (ELISA) were used to measure the oxidative stress response and downstream microglial activation and proinflammatory cytokines, and Golgi staining and long-term potentiation (LTP) recording were applied to evaluate synaptic plasticity.

**Results:**

Overexpression of SIRT3 in the CA1 region attenuated anesthesia/surgery-induced learning and memory dysfunction as well as synaptic plasticity dysfunction and the oxidative stress response (superoxide dismutase [SOD] and malondialdehyde [MDA]) in aged mice with POCD. In addition, microglia activation (ionized calcium binding adapter molecule 1 [Iba1]) and neuroinflammatory cytokine levels (tumor necrosis factor-alpha [TNF-α], interleukin [IL]-1β and IL-6) were regulated after anesthesia/surgery in a SIRT3-dependent manner.

**Conclusion:**

The results of the current study demonstrate that SIRT3 has a critical effect in the mechanism of POCD in aged mice by suppressing hippocampal neuroinflammation and reveal that SIRT3 may be a promising therapeutic and diagnostic target for POCD.

**Supplementary Information:**

The online version contains supplementary material available at 10.1186/s12974-021-02089-z.

## Background

Postoperative cognitive dysfunction (POCD) is one of the most common postoperative complications in aged patients [1]. Evidence from clinical research has revealed that POCD may contribute to increased mortality and decreased quality of life as well as prolonged hospitalization [2]. Nevertheless, the mechanism of POCD in aged patients is still unclear.

Mitochondrial damage, neuroinflammation, and synaptic plasticity dysfunction have been demonstrated to be involved in the mechanism of POCD [3–9]. Netto et al. reported oxidative damage in the hippocampus and decreased activity of the antioxidant enzyme superoxide dismutase (SOD) in aged rats with tibial fracture [10]. In addition, anesthesia/surgery may induce microglial activation, resulting in the release of inflammatory factors, such as tumor necrosis factor-alpha (TNF-α), interleukin (IL)-1β, and IL-6 [11, 12]. Furthermore, the hippocampal CA1 region is critical for cognition [13], and overactivated microglia and neuroinflammation create a neurotoxic response and cause synaptic dysfunction, resulting in cognitive dysfunction [14].

The NAD-dependent deacetylase protein Sirtuin 3 (SIRT3) is related to mitochondrial function and oxidative stress regulation [15]. SIRT3 is found mostly in the mitochondria and is the only sirtuin that specifically plays a role in extended lifespan in humans [16, 17]. Several SIRT3 substrates have been reported in the past few years that are involved in pathological mechanisms dependent on mitochondrial biogenesis and dynamism pathways [18]. It was reported that SIRT3 overexpression increased the expression of SOD2 [19]. Tyagi et al. found increased IL-1β levels and microglial activation in SIRT3^−/−^ mouse brains [20]. In addition, the mitochondria-related inflammatory response was also shown to be mediated by SIRT3 [21, 22]. SIRT3^−/−^ mice exhibited poor remote memory [23], suppressed long-term potentiation (LTP), and decreased neuronal number [23]. However, less is known about the role of SIRT3 in POCD in aged mice.

Consequently, the aim of our current study was to determine the effect of SIRT3 on anesthesia/surgery-induced cognitive impairment and the related molecular mechanism. We propose that SIRT3 may attenuate postoperative cognitive dysfunction by regulating the hippocampal neuroinflammation pathway.

## Materials and methods

### Animals

All experiments were approved by the Animal Care and Use Committee of Xuzhou Medical University and in accordance with the Guide for the Care and Use of Laboratory Animals of National Research Council. Aged (18 months old, weighing 28–32 g) male C57BL/6J mice were ordered from the Model Animal Research Center of Nanjing University. Five mice per cage were group-housed (22–25°C) with food and water available ad libitum under a 12-h light/dark cycle. The animals were randomly grouped and underwent experiments in an unbiased double-blind manner during the daytime.

### POCD mouse model

**Fig. 1:**
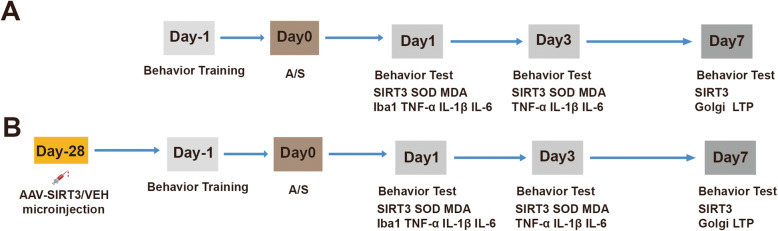
Schematic diagram of the experimental procedure. **a** The mice received behavior training at day − 1 and behavioral tests on days 1, 3, and 7 after anesthesia/surgery. Anesthesia/surgery (A/S) was performed on day 0. Hippocampal tissues were harvested at the end of the behavioral tests on days 1, 3, and 7. Western blotting analysis of SIRT3 expression was performed on days 1, 3, and 7. The levels of SOD, MDA, TNF-α, IL-1β, and IL-6 were measured on days 1 and 3. Immunofluorescence and western blotting analysis for Iba1 were performed on day 1. Golgi staining and LTP of synaptic function were measured on postoperative day 7. **b** AAV-SIRT3/VEH virus was microinjected into the CA1 region of the hippocampus 28 days before anesthesia/surgery. The mice received behavioral training at day − 1 and behavioral tests on days 1, 3, and 7 after anesthesia/surgery. Western blotting analysis of SIRT3 expression was performed on days 1, 3, and 7. The levels of SOD, MDA, TNF-α, IL-1β, and IL-6 were measured on days 1 and 3. Immunofluorescence and western blotting analysis for Iba1 were performed on day 1. Golgi staining and LTP of synaptic function were measured on postoperative day 7

Tibial fracture procedures were performed under isoflurane anesthesia to establish the POCD model as described in other studies [3]. In the first experiment, the mice were randomly placed into 2 groups: the control group (C) and the anesthesia/surgery group (A/S). The mice in group C received 100% oxygen without any anesthesia/surgery procedure [3]. For the anesthesia/surgery group, mice received 3.0% isoflurane for anesthesia induction followed by 1.5% isoflurane for maintenance. Next, the incision was made lateral to the tibia, exposing the bone. An intramedullary fixation pin was implanted into the spinal canal from the hole drilled at the trochanter of the tibia. Osteotomy was performed in the middle and distal tibia, and the incision was continuously sutured. The mouse body temperature was monitored and kept between 36 and 37°C by a heating pad during the operation. After the mice recovered from anesthesia/surgery, they were returned to their home cages. Lidocaine (2%) was locally applied to treat postoperative pain. For the interventional study, the mice were randomly divided into 4 groups: the control + control adeno-associated virus (AAV) vehicle (VEH) group (C + VEH), the anesthesia/surgery + control AAV-VEH group (A/S + VEH), the control + AAV-SIRT3 vector group (C + SIRT3), and the anesthesia/surgery + AAV-SIRT3 vector group (A/S + SIRT3). A schematic diagram of the experimental procedure is shown in Fig. 1.

### Viral microinjection

The construct was packaged into a chimeric AAV2/8 vector, and the rAAV-mNeonGreen vector without SIRT3 was used as a control AAV vehicle (AAV-VEH). The virus constructs were generated by OBiO Technology (Shanghai, China). A rAAV-CMV-SIRT3-IRES2-mNeonGreen vector (AAV-SIRT3) and a control rAAV-CMV-MCS-IRES2-mNeonGreen vehicle (AAV-VEH) (titers > 1.0 × 10^12^) were delivered by bilateral stereotactic injections into the CA1 region of the brain (0.5 μl/side) following the mouse brain atlas (AP 2.00 mm, lateral ± 1.50 mm, DV-1.40 mm). The mice were allowed to recover from the virus injection for 4 weeks before anesthesia/surgery. The location of virus transfection was confirmed by fluorescence.

### Behavioral tests

The behavioral tests included the open field test (OFT) and fear conditioning test (FCT). We used the OFT to measure the locomotor activity of the mice at different time points. The mice were allowed to freely move in the square field (40 × 40 × 40 cm) for 5 min. A video camera was used to automatically track and record movement traces of the mice with the ANY-maze software system (ANY-maze, Stoelting Co., IL, USA). The total distance traveled was used to assess the locomotor activity of the mice. The installation was wiped with 75% ethanol after each test to avoid olfactory cues.

The FCT was employed following the protocol described in previous studies [3]. During the training session before anesthesia/surgery, the mice were placed in the chamber to acclimate for 2 min, and then conditional (20-s, 70-dB tone conditional stimulus, 25-s interval) and unconditional (2-s and 0.70-mA footshock) stimuli were applied in six pairs. The intervals between the pairs were random and ranged from 45 to 60 s. For the test session, the context test was used to measure hippocampus-dependent memory, and the tone test was used to measure hippocampus-independent memory. The mice were allowed in the conditioning chamber without any stimulus for 5 min during the context test, and 2 h later, the tone test was performed. The mice were transferred into a novel chamber with the sound stimulus (70 dB, 3 min) but not the footshock stimulus for 5 min. The freezing time during each test was recorded and analyzed by the system software (Med Associates, Inc., USA).

### Western blotting analysis

Western blotting was performed as described before [24]. Briefly, we measured the protein concentrations in the CA1 region of the hippocampus by the bicinchoninic acid (BCA) Protein Assay Kit (Beyotime, P0010, Shanghai, China). Then, SDS-PAGE was used to separate the proteins, which were subsequently transferred to PVDF membranes (Merck Millipore, ISEQ00010, USA). Five percent nonfat milk with 0.1% Tween-20 in theta burst stimulation (TBS) was used to block the membranes, and the membranes were then incubated at 4 °C overnight in a cold room. The following primary antibodies were used: SIRT3 (1:1000, 5490, Cell Signaling Technology, USA), Iba1 (1:1000, ab178847, Abcam, UK), and β-actin (1:2000, AC004, ABclonal, China). Horseradish peroxidase-conjugated antibodies (1:2000, Beyotime) were used as secondary antibodies. An ECL detection system (Beyotime) and the ImageJ software were used to quantify the protein bands. The raw image files of the western blots are shown in the supplementary information (Additional file 1).

### Immunofluorescence staining of ionized calcium binding adapter molecule 1 (Iba1)

Immunofluorescence staining of the microglial marker Iba1 was used to study microglial morphology. Mice were perfused transcardially with 0.9% saline followed by 4% paraformaldehyde in 0.1 M phosphate buffer (pH 7.4). The brain tissues were removed and postfixed in 4% paraformaldehyde overnight and cryoprotected in 30% sucrose as we reported before [25]. Sections (30 μm thick) were prepared with a freezing microtome (VT1000S, Leica Microsystems). The brain tissue was blocked with 5% goat serum albumin for 30 min and then incubated with the primary antibody for Iba1 (1:100, ab178847, Abcam) in a cold room at 4 °C overnight. The brain tissue was incubated with goat anti-rabbit Alexa 594 (1:500, ab150080, Abcam) as the secondary antibody in the dark at room temperature for 2 h. The fluorescence intensity was visualized under a confocal microscope (Zeiss, LSM880, Germany). The fluorescence intensities from three slides (three visual fields per slide) were averaged for each animal and then normalized to those of the control group as we reported before [25].

### Enzyme-linked immunosorbent assay (ELISA)

Hippocampal homogenate was obtained on days 1 and 3 after anesthesia/surgery and centrifuged at 10000*g* at 4 °C for 10 min to collect the supernatant. TNF-α (Cat. No. E-EL-M0049c, Elabscience), IL-1β (Cat. No. E-EL-M0037c, Elabscience), and IL-6 (Cat. No. E-EL-M0044c, Elabscience) were measured according to the manufacturer’s protocols. The optical density (OD) at 450 nm was obtained with an ELISA plate reader (Multiskan GO, Thermo Scientific, USA).

### Oxidative activity evaluation

SOD and malondialdehyde (MDA) were measured by spectrophotometric assay kits (Elabscience, Wuhan, China). A Varioskan LUX microplate reader was used to determine SOD activity by measuring the absorbance at 450 nm (U/mg protein), and MDA activity was determined by measuring the absorbance at 532 nm (nmol/mg protein).

### Golgi-Cox staining and dendritic spine counting

Golgi-Cox staining was used to visualize synaptic structural plasticity. The HITO Golgi-Cox OptimStain PreKit (Hitobiotec Corp, Kingsport, TN, USA) was employed according to the manufacturer’s instructions. Specifically, the brain tissues were immersed in solutions 1 and 2 for 24 h, the solutions were changed, and the tissues were stored in the dark at room temperature for 2 weeks. Next, solution 3 was used, and the brains were stored in the dark at 4 °C for another 72 h. A cryotome (chamber temperature − 22 °C) was used to prepare brain sections (100 μm). An Olympus BX53 was used for the imaging and analysis of the dendrites and dendritic spines within the CA1 region of the hippocampus.

### Multichannel field potential recording

LTP recordings were used to evaluate functional synaptic plasticity, and the 64-channel recording system (MED64, Panasonic Alpha-Med Sciences) was used as described previously [26, 27]. One of 64 microelectrodes was selected as a stimulating electrode within the CA1 region of the hippocampus. The evoked field excitatory postsynaptic potentials (fEPSPs) were used for recording from the other channels. Theta burst stimulation (TBS) (5 trains of bursts with 4 pulses at 100 Hz, repeated 5 times at 10-s intervals) was used in this study. Averaged fEPSP slopes over the last 20 min of the recording were used for analysis.

### Statistical analysis

Statistical analyses were performed by GraphPad Prism 6.0 (GraphPad Software, Inc.). Data are presented as the mean ± SEM. The differences between the C and A/S groups were compared by unpaired *t* test. Behavioral and other tests comprising four groups were analyzed by one-way ANOVA with a Tukey post hoc test for multiple comparisons. A significant difference was defined as *p* < 0.05.

## Results

### Effects of anesthesia/surgery on the OFT and FCT of aged mice

**Fig. 2:**
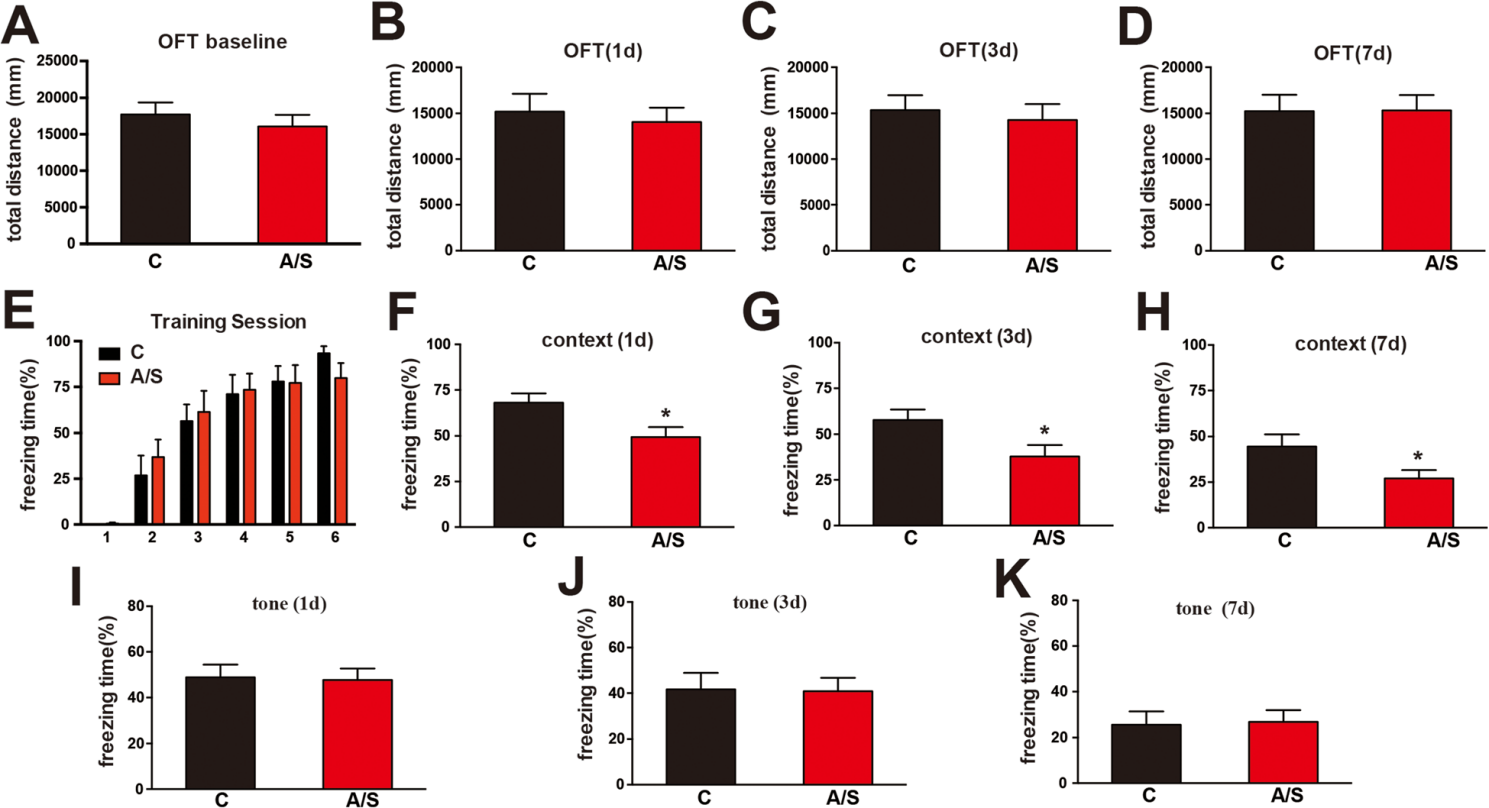
Hippocampus-dependent memory, but not hippocampus-independent memory or locomotor activity, in mice was impaired by anesthesia/surgery. The OFT was conducted to assess the total distance traveled by the mice at 1 day preoperatively and at postoperative days 1, 3, and 7 (**a**–**d**). The percent of freezing time during the training session at 1 day preoperatively (**e**) in the context test (**f**–**h**) and tone test (**i**–**k**) were assessed at postoperative days 1, 3, and 7. The data are presented as the means ± standard error of the mean for each group (*n* = 10). *p < 0.05 compared with the control group

The OFT was employed to assess the locomotor activity of aged mice. We found that the total distance traveled by the mice was not significantly changed between the control (C) and anesthesia/surgery (A/S) groups at baseline (1 day before anesthesia/surgery) (*p* > 0.05, Fig. 2a) or postoperation (1, 3, and 7 days after anesthesia/surgery) (*p* > 0.05, Fig. 2b–d). In the FCT, context tests were used to examine hippocampus-dependent learning and memory, while tone tests were used to examine hippocampus-independent learning and memory [3]. During the training sessions, we found that the freezing time was not significantly different between the C and A/S groups (*p* > 0.05, Fig. 2e), which means that the cognitive condition of the aged mice was comparable before the operation. In the FCT context test, the freezing time was significantly decreased at 1, 3, and 7 days after anesthesia/surgery in the A/S group compared to the C group (*p < 0.05, Fig. 2f–h). Nevertheless, in the FCT tone test, the freezing time of the A/S group did not obviously differ from that of the C group after the operation (*p* > 0.05, Fig. 2i–k). Taken together, these results indicated that anesthesia/surgery induced hippocampus-dependent cognitive decline but did not induce hippocampus-independent cognitive decline or locomotor activity impairment in aged mice.

### Effects of anesthesia/surgery on SIRT3 expression and the mitochondrial oxidative stress response in the hippocampus of aged mice

**Fig. 3:**
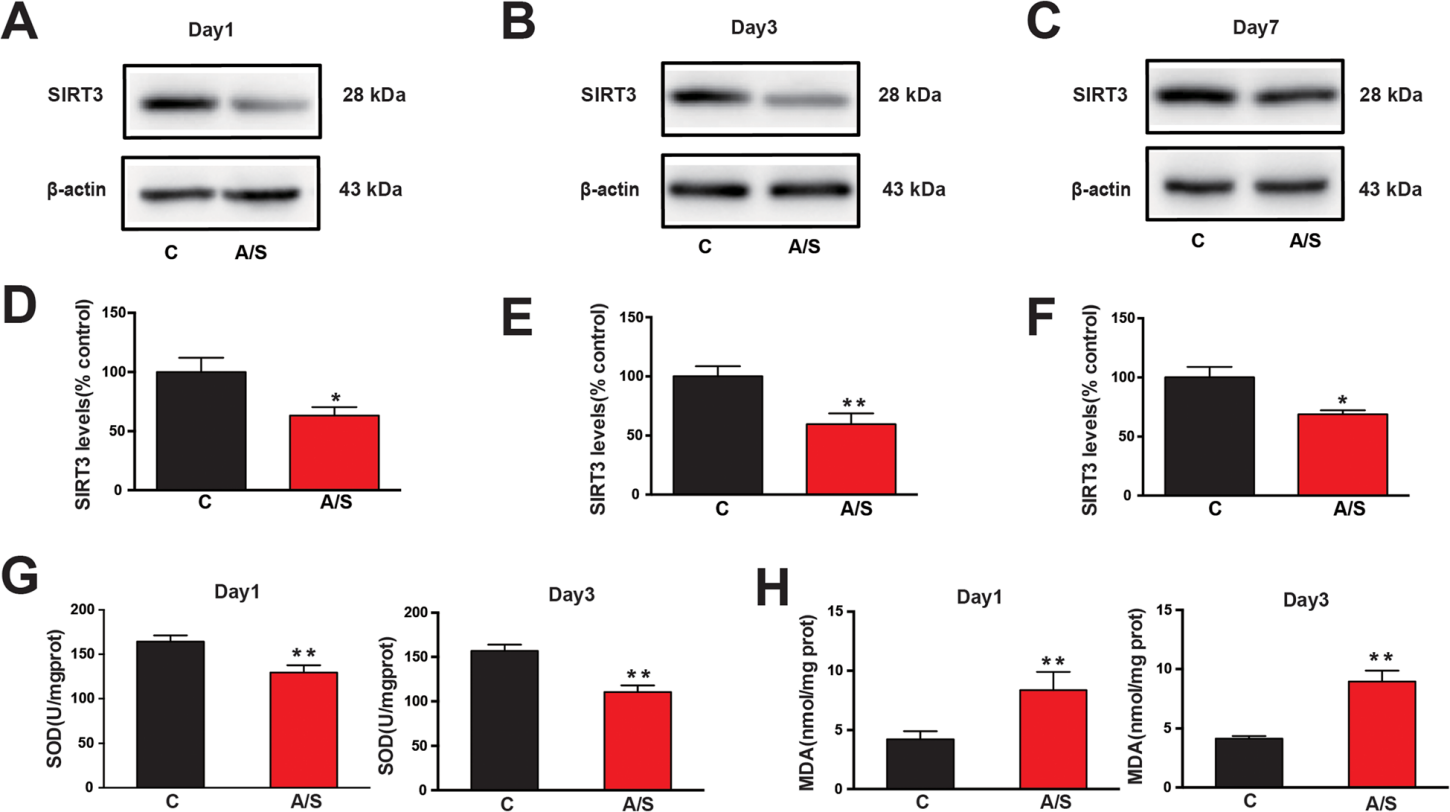
SIRT3, SOD, and MDA levels in the hippocampal CA1 region of aged mice after anesthesia/surgery. Representative western blots of SIRT3 at days 1, 3, and 7 after anesthesia/surgery (**a**–**c**). Quantitative analysis of SIRT3 levels compared with the control group (**d**–**f**). Analysis of SOD (**g**) and MDA (**h**) levels in the hippocampal CA1 region on postoperative days 1 and 3. The data are presented as the means ± standard error of the mean for each group (*n* = 6). *p < 0.05, **p < 0.01 compared with the control group

SIRT3 expression levels in the CA1 region of the hippocampus were measured in the control and anesthesia/surgery groups. Compared with that in the control mice, the SIRT3 expression in the A/S mice was obviously decreased at postoperative days 1, 3, and 7 (*p < 0.05, Fig. 3a–f). To evaluate the mitochondrial oxidative stress response, the levels of SOD and MDA in the CA1 region of aged mice were determined by ELISA. Anesthesia/surgery induced decreased SOD levels in the hippocampal CA1 region on postoperative days 1 and 3 in aged mice (*p < 0.05, Fig. 3g). Meanwhile, anesthesia/surgery significantly increased the level of MDA in the CA1 region of the hippocampus compared with the control group (*p < 0.05, Fig. 3h). These results showed that anesthesia/surgery induced the downregulation of SIRT3 and increased the oxidative stress response in the CA1 region of the hippocampus of aged mice.

### Effects of anesthesia/surgery on hippocampal microglia activation and neuroinflammation in aged mice with POCD

**Fig. 4:**
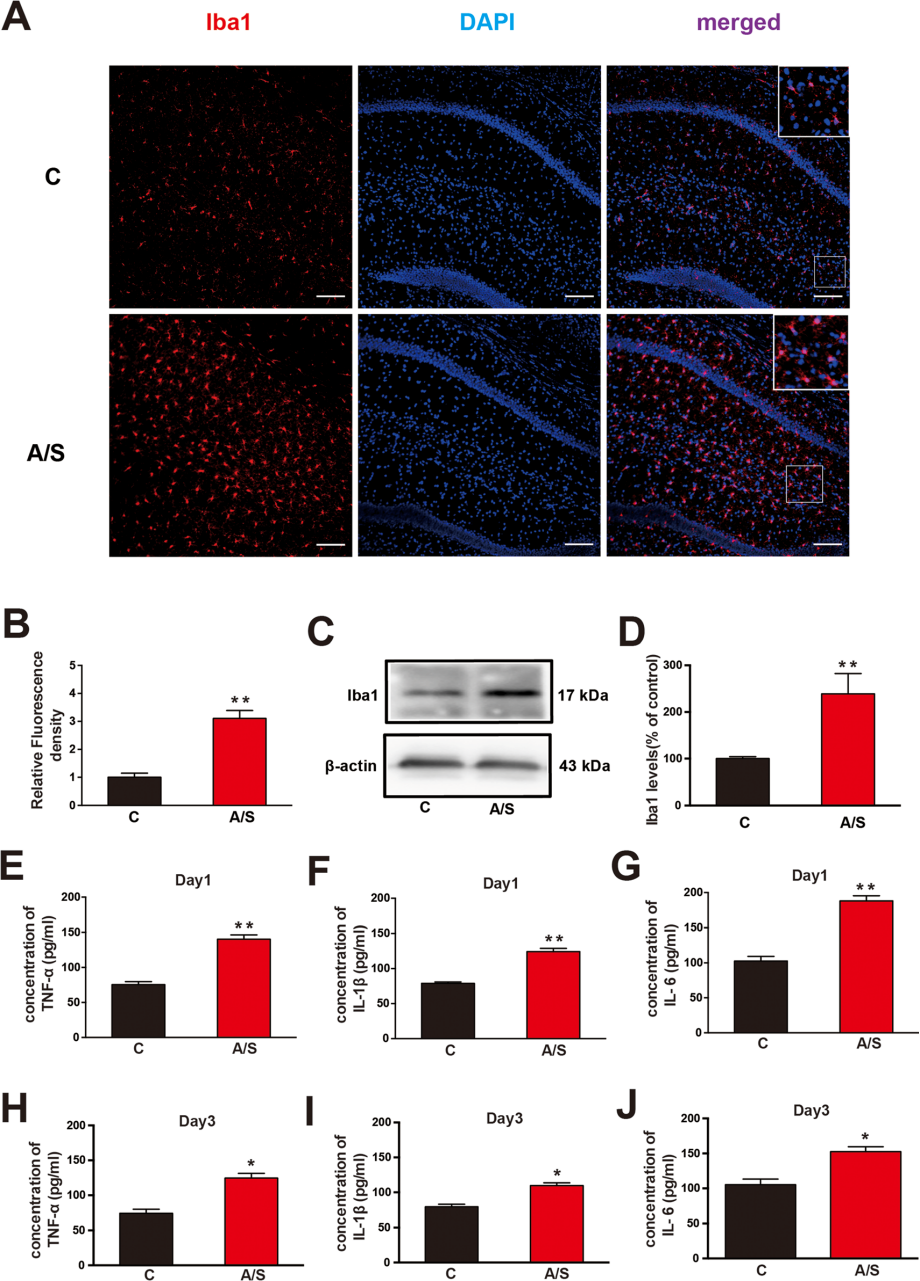
Anesthesia/surgery induced microglial activation and neuroinflammation in the hippocampus of aged mice. **a** Representative images of Iba1 (a marker of microglia) in the CA1 region at day 1 after anesthesia/surgery. **b** Quantification of Iba1 fluorescence. **c** Representative western blots of Iba1 at day 1 after anesthesia/surgery. **d** Quantitative analysis of Iba1 levels compared with the control group. Analysis of TNF-α, IL-1β, and IL-6 levels in the hippocampal CA1 region on postoperative days 1 (**e**–**g**) and 3 (**h**–**j**). The data are presented as the means ± standard error of the mean for each group (*n* = 6). *p < 0.05, **p < 0.01 compared with the control group

Microglia activation is an essential early-phase change in hippocampal neuroinflammation in POCD. First, we found by immunofluorescence staining that Iba1 expression was increased in the CA1 region of the hippocampus on postoperative day 1 (**p < 0.01, Fig. 4a, b). Correspondingly, immunoblotting for Iba1 revealed a visible increase in Iba1 expression in the CA1 region of the hippocampus of aged mice on postoperative day 1 (**p < 0.01, Fig. 4c, d). Then, the TNF-α, IL-1β and IL-6 levels were used to assess the level of neuroinflammation in aged mice. As shown in Fig. 4e–j, the levels of TNF-α, IL-1β, and IL-6 in the CA1 were increased in the anesthesia/surgery group compared with the control group on postoperative days 1 and 3 (*p < 0.05). These data indicated that anesthesia/surgery induced microglia activation and neuroinflammation in the CA1 region of the hippocampus of aged mice.

### Effects of anesthesia/surgery on synaptic plasticity in the hippocampus of aged mice with POCD

**Fig. 5:**
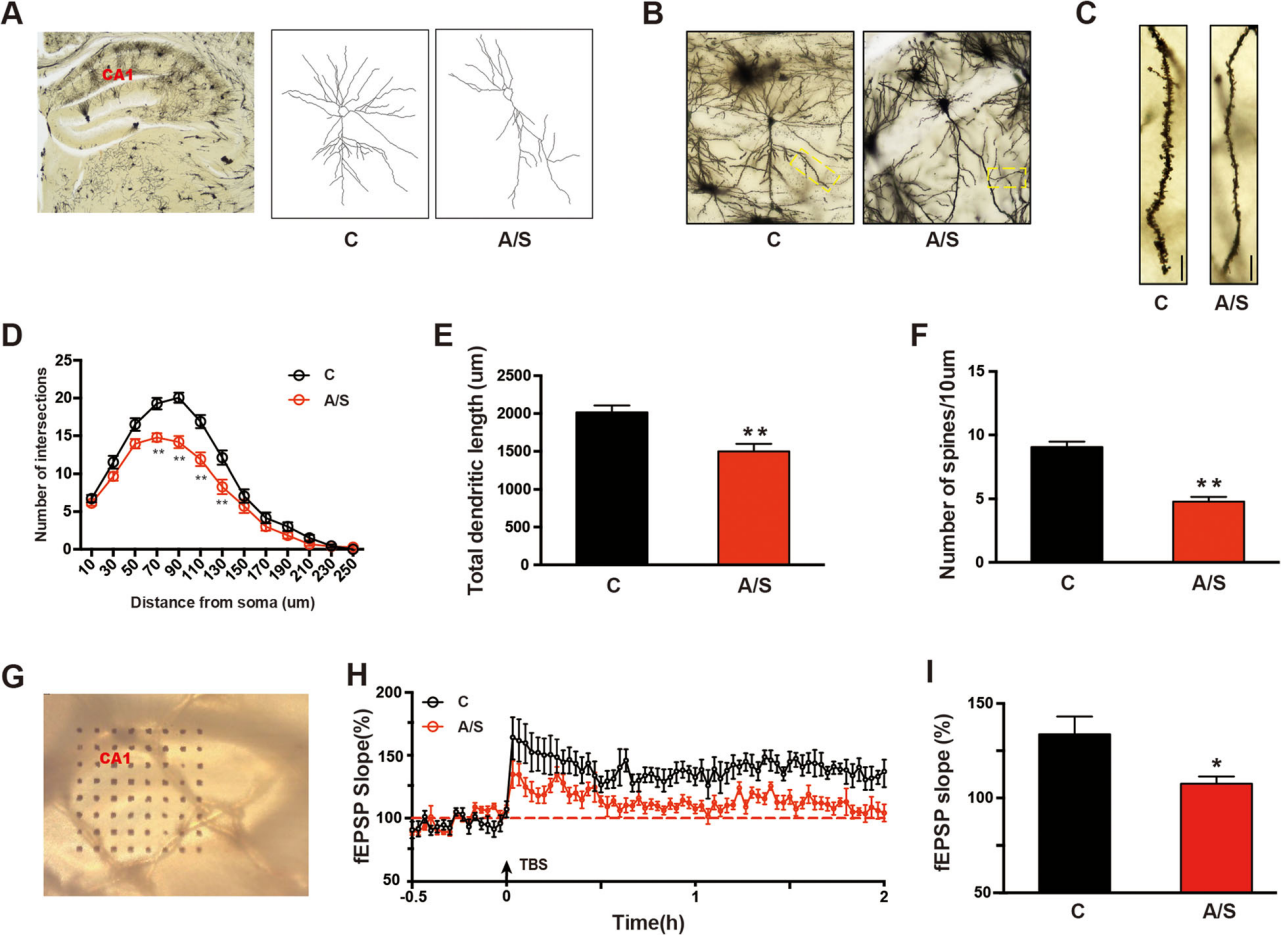
Hippocampal synaptic plasticity changes on day 7 after anesthesia/surgery. **a** A hippocampal profile image of Golgi staining and camera tracings of hippocampal CA1 neurons. **b** Sample images of Golgi-Cox staining of CA1 neurons for spine counting. **c** Representative dendritic spine density of hippocampal CA1 neurons. **d** Quantitation of dendritic intersections (*n* = 18). **e** Quantitation of the total dendritic length (*n* = 18). **f** Quantitation of the dendritic spine density (*n* = 18). **g** Sample images showing the location of the MED64 probe in the CA1 region. **h** LTP recording in the hippocampal CA1 region. Arrows indicate the time point of TBS application. I. Averaged fEPSP slope during the last 20 min. The data are presented as the means ± standard error of the mean for each group (*n* = 6). *p < 0.05, **p < 0.01 compared with the control group. Scale bar = 10 μm

Previous studies have revealed that synaptic plasticity is a critical molecular mechanism for learning and memory [28, 29]. First, synaptic structural plasticity was investigated by Golgi-Cox staining. The morphology of the CA1 neurons and camera tracings from the control and anesthesia/surgery groups are shown in Fig. 5a. Sholl analysis was used to measure dendritic branching and spine density in the CA1 region of the hippocampus. The total number of dendritic intersections at 70–130 μm from the soma was significantly reduced in the anesthesia/surgery group (**p < 0.01, Fig. 5d). Additionally, the total dendritic length and number of spines were also reduced after anesthesia/surgery (**p < 0.01, Fig. 5b, c and e, f). Then, synaptic functional plasticity was measured by electrophysiological methods. LTP in the CA1 region of the hippocampus was evaluated using a 64-channel multielectrode (MED64) system. As shown in Fig. 5g–i, LTP induced by TBS in the CA1 region of aged mice was attenuated in the A/S group on postoperative day 7. These results indicated that anesthesia/surgery induced both structural and functional synaptic plasticity dysfunction in the CA1 region of the hippocampus of aged mice.

### SIRT3 overexpression in the hippocampal CA1 region of aged mice with POCD

**Fig. 6:**
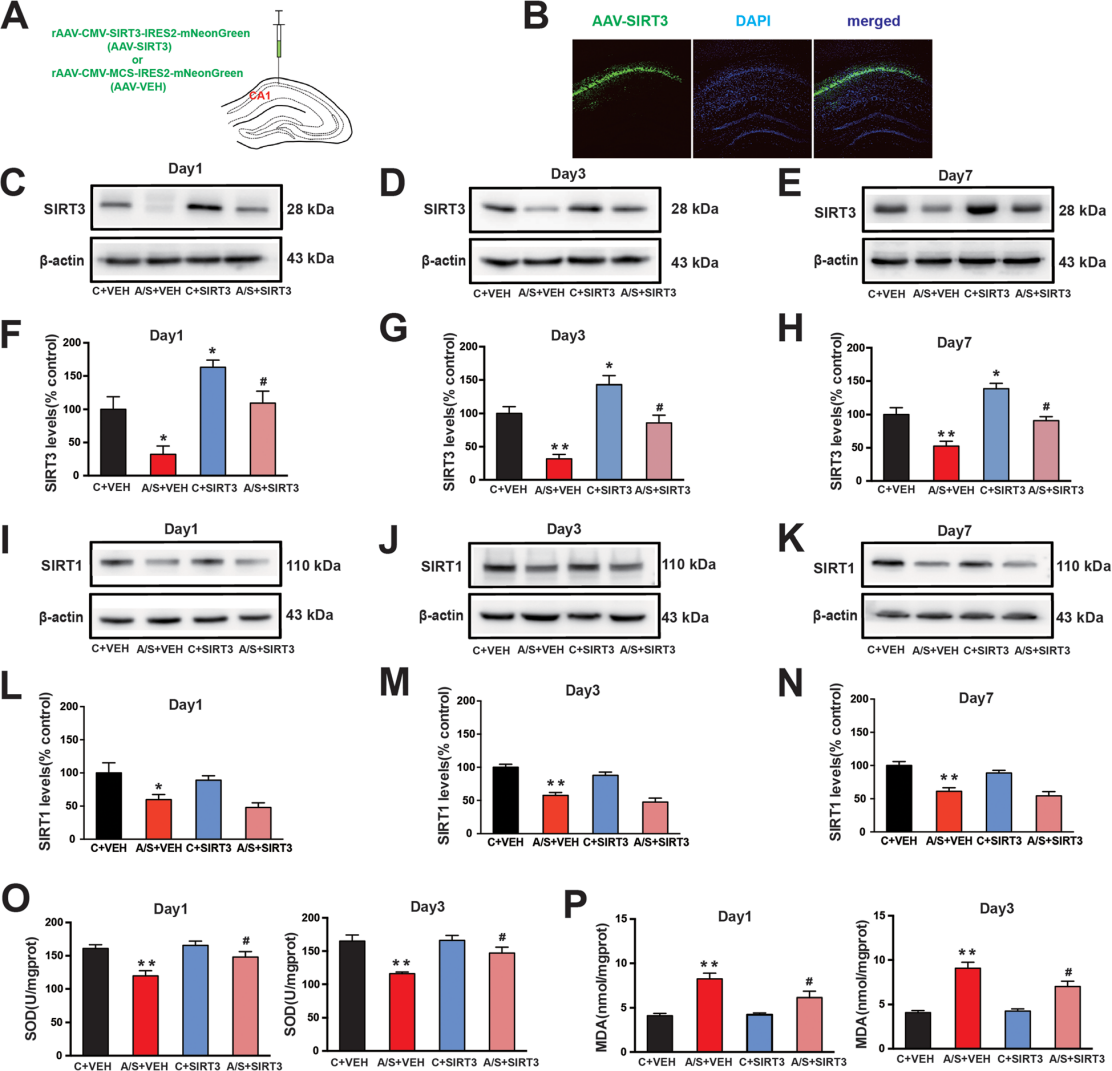
Overexpression of SIRT3 in CA1 region of aged mice. **a** The location for viral microinjection with a rAAV-CMV-SIRT3-IRES2-mNeonGreen vector (AAV-SIRT3) or a control rAAV-CMV-MCS-IRES2-mNeonGreen vehicle (AAV-VEH). **b** Fluorescence images showing efficient expression of AAV-SIRT3 vector in CA1 region. Representative images of Western blots showing that AAV-SIRT3 injection increased SIRT3 expression in CA1 cells (**c**–**e**). Quantification of SIRT3 expression among the 4 groups (**f**–**h**). Representative images of Western blots and quantification of SIRT1 expression among the 4 groups (**i**–**n**). Analysis of SOD and MDA levels in the hippocampal CA1 region on postoperative days 1 and 3 (**o**, **p**). The data are presented as the means ± standard error of the mean for each group (*n* = 6). *p < 0.05, **p < 0.01 compared with the C + VEH group, ^#^*p* < 0.05 compared with the A/S + VEH group

To demonstrate the role and mechanism of SIRT3 in POCD in aged mice, AAV-SIRT3 vector (which carries the SIRT3 gene) was locally injected into the CA1 region of aged mice 4 weeks before anesthesia/surgery, and AAV-VEH vector was injected as a control (Fig. 6a, b). The SIRT3 expression level was increased in the C + SIRT3 group but decreased in the A/S + VEH group compared with that of the control group, while the overexpression of SIRT3 rescued the decreased SIRT3 level in the A/S + SIRT3 group compared with that of the A/S + VEH group at postoperative days 1, 3, and 7 of aged mice (*p* < 0.05, Fig. 6c–h). As shown in Fig. 6i–n, SIRT1 expression was markedly decreased after anesthesia/surgery. However, the expression level of SIRT1 in the A/S + SIRT3 group was not statistically different from that in the A/S + VEH group on postoperative days 1, 3, and 7. Similarly, there was also no statistic difference in SIRT1 expression between the C + SIRT3 group and the C + VEH group. Then, the SOD and MDA levels were measured in the 4 groups. We found that the SOD level was significantly decreased in the A/S + VEH group compared with the control group on postoperative days 1 and 3 (*p* < 0.05, Fig. 6o). Meanwhile, the level of SOD in the A/S + SIRT3 group was significantly increased compared with that in the A/S + VEH group. Conversely, compared with that of the C + VEH group, the level of MDA (*p* < 0.05, Fig. 6p) was significantly increased in the A/S + VEH group on postoperative days 1 and 3. Furthermore, preoperative injection of AAV-SIRT3 in the CA1 significantly attenuated the upregulation of MDA. These data indicated that the anesthesia/surgery-induced mitochondrial oxidative stress response in the CA1 region of the hippocampus of aged mice with POCD was attenuated in a SIRT3-dependent manner.

### Effects of SIRT3 overexpression on microglia activation and neuroinflammation in the hippocampus of aged mice with POCD

**Fig. 7:**
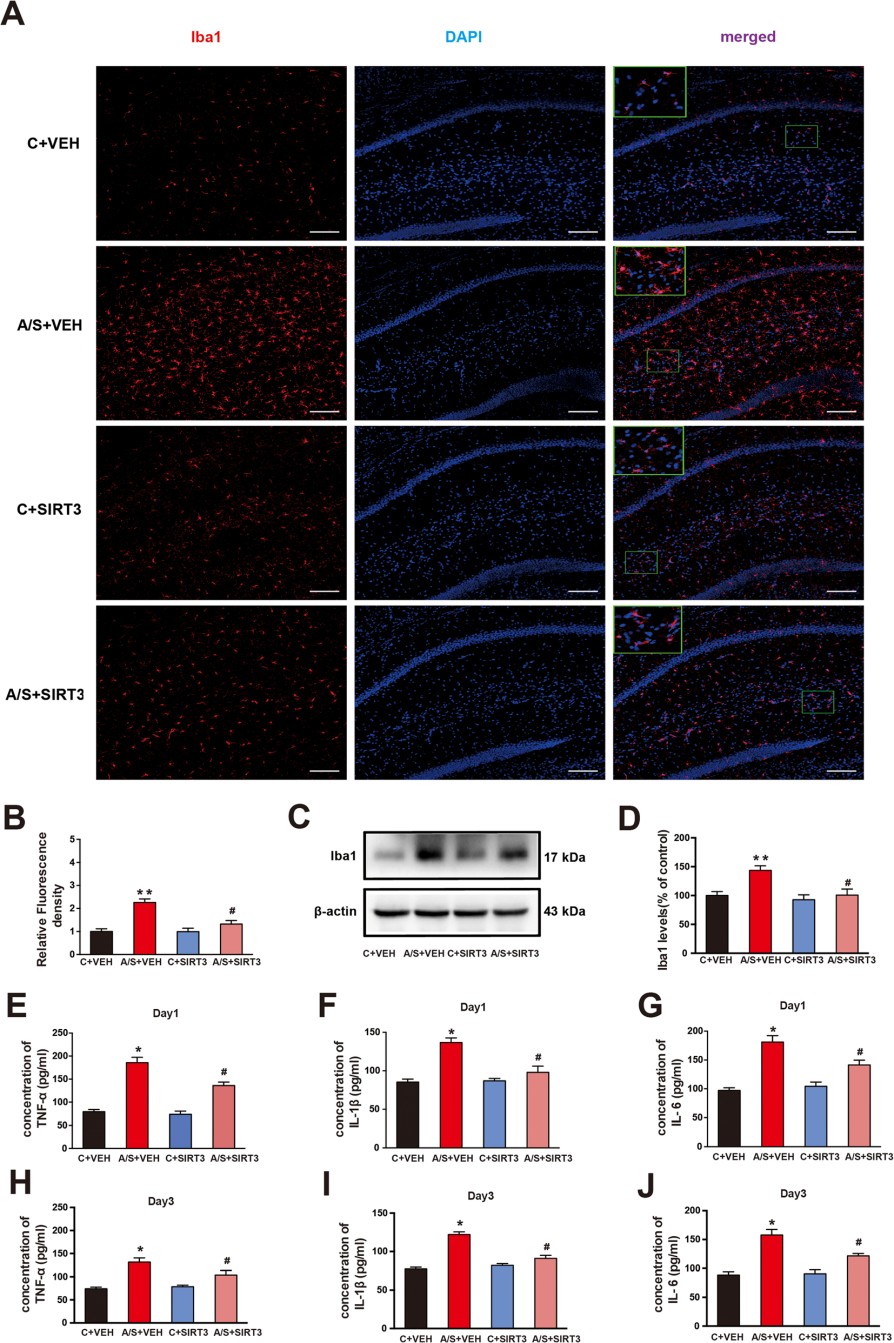
Microglial activation and neuroinflammation in the hippocampal CA1 region among the 4 groups. **a** Representative images of Iba1 (a marker of microglia) in the CA1 region. **b** Quantification of Iba1 fluorescence. **c** Representative Western blots of Iba1 among the 4 groups at day 1 after anesthesia/surgery. **d** Quantitative analysis of Iba1 levels compared with the control group. Analysis of TNF-α, IL-1β, and IL-6 levels in the hippocampal CA1 region on postoperative days 1 and 3 (**e**–**g** and **h**–**j**). The data are presented as the means ± standard error of the mean for each group (*n* = 6). *p < 0.05, **p < 0.01 compared with the C + VEH group, ^#^*p* < 0.05 compared with the A/S + VEH group

To determine the downstream signaling pathway and molecules involved in SIRT3-mediated POCD in aged mice, we detected microglia activation and neuroinflammation in the 4 groups by both immunofluorescence and western blotting. We found obviously increased fluorescence intensity and protein expression of Iba1 in the A/S + VEH group compared to the C + VEH group (**p < 0.01, Fig. 7a–d). Of note, the A/S + SIRT3 group had significantly reduced fluorescence intensity and protein expression of Iba1 compared to those of the A/S + VEH group (Fig. 7a–d, ^#^*p* < 0.05). Next, the TNF-α, IL-1β, and IL-6 levels were measured in the 4 groups. Compared with those in the C + VEH group, the levels of TNF-α, IL-1β, and IL-6 (*p* < 0.05, Fig. 7e–j) were significantly increased in the A/S + VEH group on postoperative days 1 and 3. Furthermore, preoperative injection of AAV-SIRT3 in the CA1 significantly attenuated the upregulation of TNF-α, IL-1β, and IL-6 levels. Thus, these data indicated that microglia activation and neuroinflammation may be involved in the pathogenesis of SIRT3-mediated POCD in aged mice.

### Effects of SIRT3 overexpression on synaptic plasticity in the hippocampus of aged mice with POCD

**Fig. 8:**
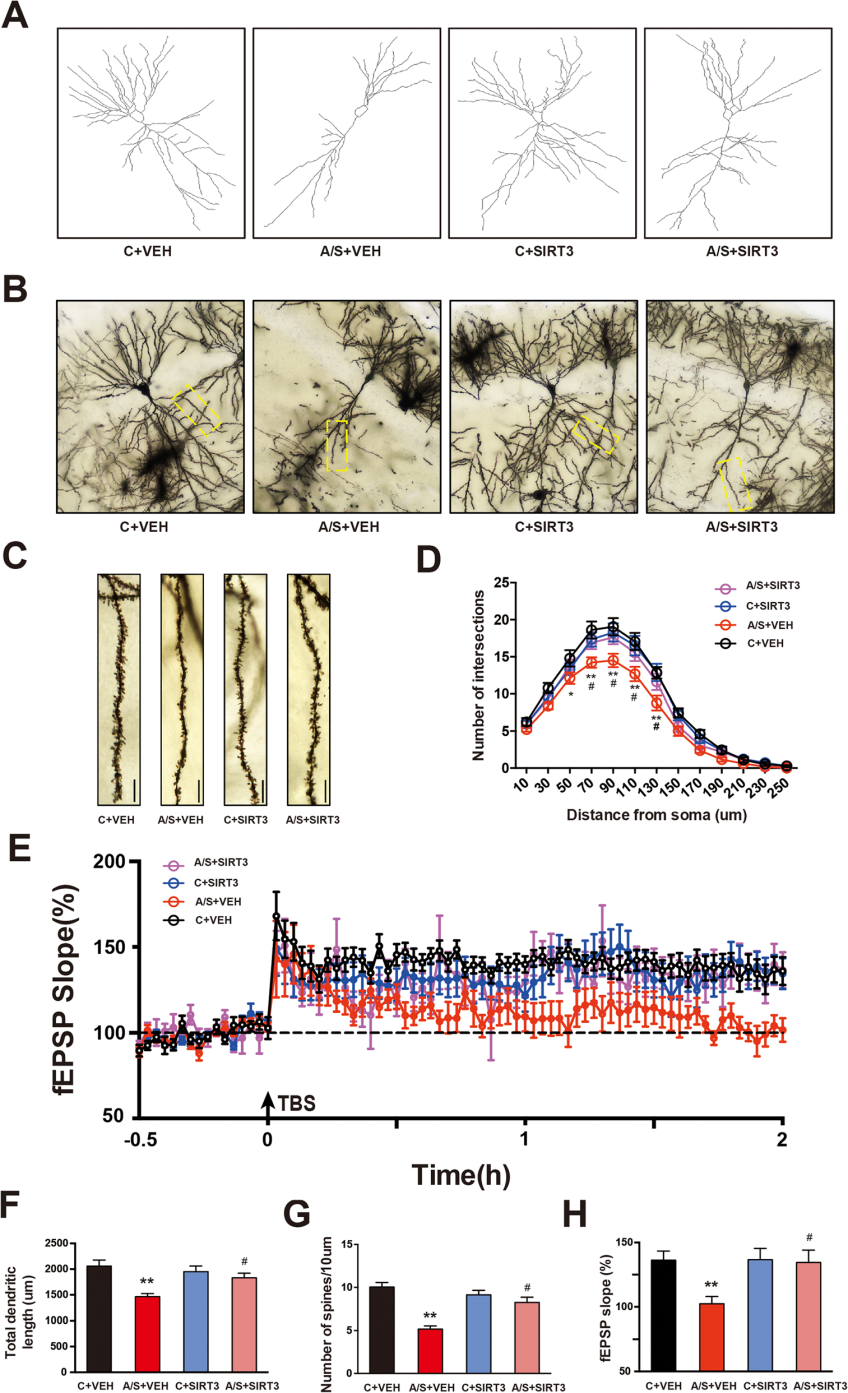
Hippocampus synaptic plasticity among the 4 groups. **a** Camera tracings of hippocampal CA1 neurons among the different groups. **b** Sample images of Golgi-Cox staining of CA1 neurons for spine counting. **c** Representative dendritic spine density of hippocampal CA1 neurons. **d** Quantitation of the dendritic intersections. **e** Quantitation of the total dendritic length. **f** Quantitation of the dendritic spine density. **g** LTP recording in the hippocampal CA1 region. Arrows indicate the time point of TBS application. **h** Averaged fEPSP slope during the last 20 min. The data are presented as the means ± standard error of the mean for each group (*n* = 6). *p < 0.05, **p < 0.01 compared with the C + VEH group, ^#^*p* < 0.05 compared with the A/S + VEH group. Scale bar = 10 μm

Regarding synaptic structural plasticity, Sholl analysis showed that the total number of dendritic intersections at 50–130 μm from the soma in the hippocampal CA1 region was reduced significantly in the A/S + VEH group compared to the C + VEH group (*p < 0.05, Fig. 8a, d). SIRT3 overexpression significantly upregulated the total number of dendritic intersections in the A/S + SIRT3 group compared to the A/S + VEH group (^#^*p* < 0.05, Fig. 8a, d). Additionally, SIRT3 overexpression also significantly upregulated the total dendritic length and number of spines in the A/S + SIRT3 group compared to the A/S + VEH group (^#^*p* < 0.05, Fig. 8b, c, e–f). Regarding synaptic functional plasticity, as shown in Fig. 8g, h, LTP in the CA1 region of aged mice was suppressed in the A/S + VEH group compared to the C + VEH group on postoperative day 7, but SIRT3 overexpression significantly attenuated the loss of LTP in the A/S + SIRT3 group compared to the A/S + VEH group (Fig. 8g, h). These results indicated that SIRT3 overexpression may protect aged mice from anesthesia/surgery-induced synaptic plasticity dysfunction.

### Effects of SIRT3 overexpression on the OFT and FCT of aged mice with POCD

**Fig. 9:**
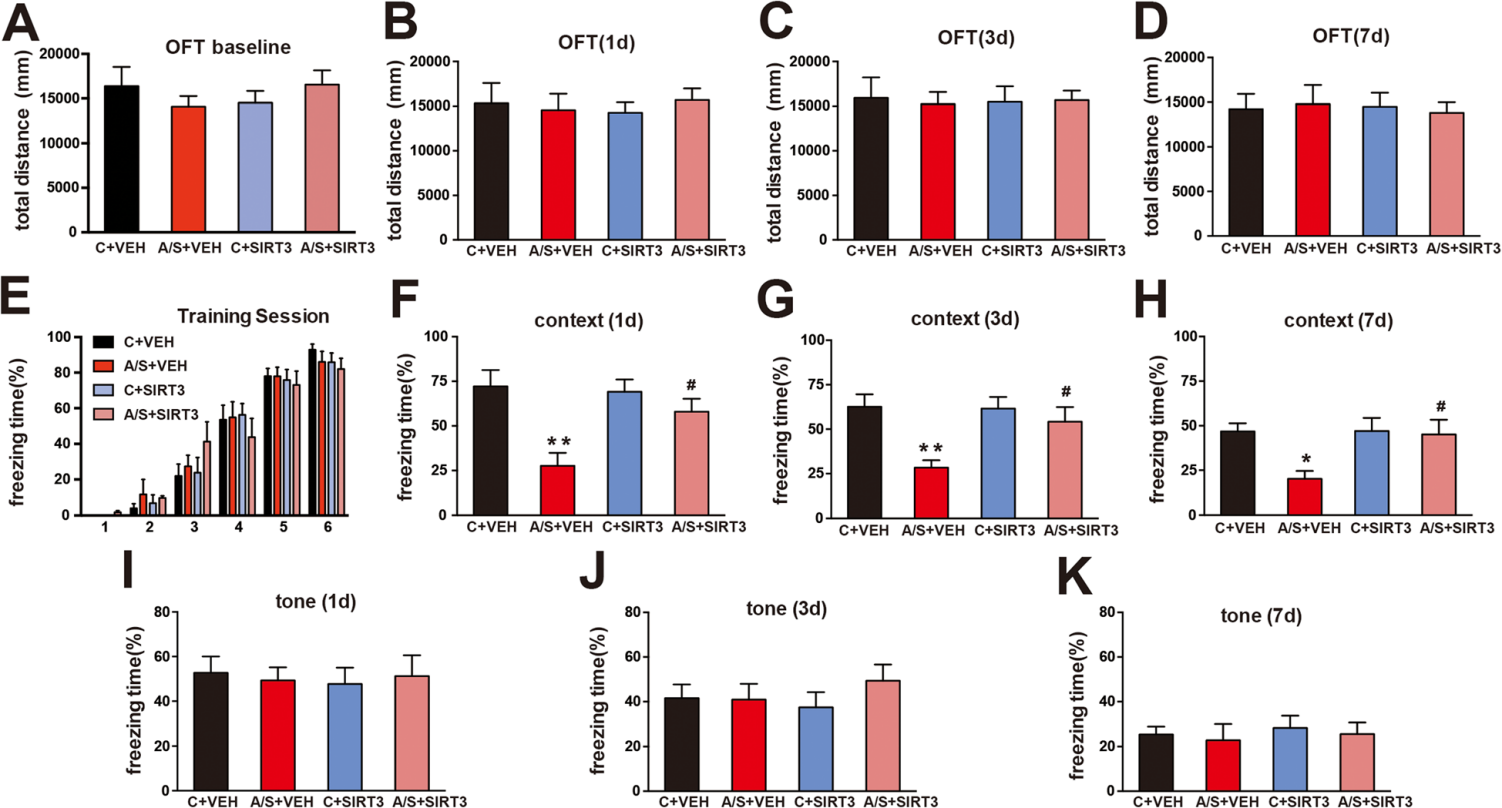
SIRT3 overexpression alleviated anesthesia/surgery-induced hippocampus-dependent cognitive dysfunction in aged mice. The total distance traveled in the OFT among the 4 groups at 1 day before and 1, 3, and 7 days after anesthesia/surgery (**a**–**d**). The percent freezing time was assessed during the training session on 1 day preoperatively (**e**) as well as in the context test (**f**–**h**) and tone test (**i**–**k**) at postoperative days 1, 3, and 7. The data are presented as the means ± standard error of the mean for each group (*n* = 10). *p < 0.05, **p < 0.01 compared with the C + VEH group, ^#^*p* < 0.05 compared with the A/S + VEH group

In the OFT, the total distance traveled was not significantly different among the 4 groups during the preoperative (*p* > 0.05, Fig. 9a) or postoperative period (*p* > 0.05, Fig. 9b–d). These results showed that the locomotor ability of the mice was not impaired by the viral injection prior to the operation or by the anesthesia/surgery procedure. In the FCT training session, no significant difference in freezing time was found among the 4 groups of aged mice (*p* > 0.05, Fig. 9e), showing that their cognition was comparable before the anesthesia/surgery procedure. Next, we found that the freezing time was decreased on postoperative days 1, 3, and 7 in the A/S + VEH group compared to the C + VEH group in the context test (*p < 0.05, Fig. 9f–h). Moreover, SIRT3 overexpression increased the freezing time during the postoperative period (postoperative days 1, 3, and 7) in the A/S + SIRT3 group compared to the A/S + VEH group in the context test (^#^*p* < 0.05, Fig. 9d–f). Additionally, the freezing time was not significantly different among the 4 groups on postoperative days 1, 3, and 7 in the tone test (*p* > 0.05, Fig. 9i–k). Collectively, these results indicated that SIRT3 overexpression attenuated anesthesia/surgery-induced hippocampus-dependent cognitive decline in aged mice.

## Discussion

The purpose of the current study was to assess the effect of SIRT3 in anesthesia/surgery-induced cognitive decline in aged mice with hippocampal inflammation-related molecular mechanisms. We found that SIRT3 expression was obviously decreased in the hippocampal CA1 region of aged mice with POCD. In addition, SIRT3 overexpression prevented anesthesia/surgery-induced cognitive decline and synaptic plasticity dysfunction in aged mice. Importantly, SIRT3 overexpression ameliorated the anesthesia/surgery-induced mitochondrial oxidative stress response, microglia activation and neuroinflammation. Hence, these results demonstrated the critical effect of SIRT3 in the treatment of anesthesia/surgery-induced neuroinflammation and cognitive dysfunction in aged mice.

Studies on patients with cognitive decline after anesthesia/surgery have been conducted for more than 100 years. In 2018, the term “perioperative neurocognitive disorders” (PNDs) was recommended by the Nomenclature Consensus Working Group as an overarching term for cognitive impairments identified in the preoperative or postoperative period [30–34], which includes cognitive decline diagnosed before operation, acute postoperative delirium, and delayed neurocognitive recovery. Since we focused on postoperative cognitive function, the term “POCD” was used in our current study.

The mitochondrial oxidative stress response contributes to postoperative cognitive dysfunction [10]. Recently, SIRT3 has been shown to be strongly involved in the regulation of oxidative stress-related mitochondrial damage in neurodegenerative diseases, such as Parkinson’s disease (PD) and Alzheimer’s disease (AD) [35]. For example, SIRT3 has been shown to be downregulated in AD patients [36]. Apolipoprotein E4 (APOE4) is a major genetic factor related to late-onset AD, and SIRT3 has been reported to be decreased in the frontal cortices of APOE4-carrying individuals compared to non-APOE4-carrying individuals [17]. Additionally, it has been reported that SIRT3 overexpression suppressed oxidative stress and therefore extended neuronal longevity in primary hippocampal cultures [37]. This suggests that SIRT3 upregulation can reverse brain oxidative stress, which is implicated in cognitive decline. Specifically, SIRT3 is known to regulate the activity of mitochondrial antioxidant enzymes. Therefore, the levels of SOD and MDA were measured in this study, and we found decreased SOD and increased MDA levels after anesthesia/surgery that could be ameliorated by SIRT3 overexpression. These findings suggest that the oxidative stress response of hippocampal mitochondria in aged mice with POCD is likely to be SIRT3-dependent. To address the potential impact of SIRT3 overexpression on other Sirt proteins, we then measured the expression level of SIRT1, another important Sirt protein in the nervous system. SIRT1 expression was markedly decreased after anesthesia/surgery, which was consistent with the results of previous studies [38, 39]. We also found that the expression level of SIRT1 in the A/S + SIRT3 group was not significantly changed compared with that in the A/S + VEH group on days 1, 3, and 7 after anesthesia/surgery. Similarly, the expression level of SIRT1 in the C + SIRT3 group was also not markedly different from that in the C + VEH group. These results indicated that the overexpression of SIRT3 did not significantly change the SIRT1 expression level, which suggested that SIRT1 may not be involved in the SIRT3-mediated neuroprotective effect in POCD. Verma et al. reported that SIRT3 KO might cause a compensatory rise in SIRT1 expression, which was responsible for neuroprotection in a stroke model [40]. However, we did not find the corresponding result that SIRT3 overexpression causes a compensatory decrease in SIRT1 expression. The different changes in SIRT1 expression (compensatory rise vs no change) may be due to the different disease models (stroke vs POCD) and the different techniques used (genetic knockout vs virus injection). Further study is still needed to fully clarify the potential interaction of SIRT3 and other Sirt proteins in POCD.

The enzymes of the redox pathway maintain the balance between the production and elimination of oxygen radicals by a well-regulated system [41]. Uncontrolled oxidative stress promotes the inflammatory response [42]. We found that the levels of the inflammatory mediators TNF-α, IL-1β, and IL-6 in the CA1 region were significantly increased at postoperative days 1 and 3 and that these changes were accompanied by changes in SOD and MDA levels, whereas SIRT3 overexpression attenuated these phenomena, suggesting a potential link between the mitochondrial oxidative stress response and neuroinflammation. Previous studies have focused on the mechanism by which cytokines generated in the periphery cross the blood–brain barrier (BBB) and induce secondary inflammation in the brain [43, 44]. We propose a mechanism in which neuroinflammation is triggered through SIRT3-dependent mitochondrial damage in the brain. Moreover, local SIRT3 overexpression in the hippocampal CA1 region ameliorated the anesthesia/surgery-induced neuroinflammation and thus confirmed the causal relationship.

In the present study, we also observed the postoperative proliferation of microglia in the CA1 region of aged mice. These results are consistent with previous studies. Anesthesia/surgery can induce the innate immune system response [45] and microglia activation, leading to the synthesis and release of inflammatory cytokines (e.g., TNF-α, IL-1β, and IL-6) [46, 47] and cognitive decline [48]. It was recently reported that pharmacological activation of SIRT3 significantly reduced the susceptibility of microglial cells to inflammatory stress [49]. Consistent with these results, preoperative injection of AAV-SIRT3 in the CA1 region attenuated microglial activation and hippocampal neuroinflammation. Therefore, these findings also indicated that microglia activation and hippocampal neuroinflammation are involved in the effects of SIRT3 in POCD in aged mice.

Microglial synaptic pruning contributes to neuronal network establishment [50]. Microglia serve as housekeepers and constantly monitor the brain environment. Microglia contact with synapses is dynamic. Microglia activation and neuroinflammation are also involved in aging-related cognitive decline and the progression of neurodegenerative diseases, including AD [51]. Furthermore, alterations in microglia activation and neuroinflammation have been associated with synaptic plasticity dysfunction in neurodegenerative diseases, such as PD [52]. Previous studies have demonstrated that the structural and functional synaptic plasticity of the hippocampus plays a critical role in the development of neurological disorders [53, 54]. It has been shown that the density of dendritic spines is impaired and the maintenance of LTP is suppressed in the hippocampus of AD mice [29, 55–57]. It has also been suggested that the density of dendritic spines of CA1 pyramidal neurons is strongly involved in memory and learning [58–60]. Qiu et al. reported anesthesia/surgery-induced changes in structural synaptic plasticity with increased neuroinflammation in aged mice by Golgi staining [4], while we measured functional synaptic plasticity by LTP recording as well. LTP, which is an increase in synaptic efficacy following high-frequency stimulation, is widely considered a molecular model for learning and memory [61–63]. LTP is also involved in the local remodeling of dendritic spines and synapses [28, 64]. We found that both structural and functional synaptic plasticity in the CA1 region were impaired after anesthesia/surgery in aged mice, while SIRT3 overexpression reversed these synaptic changes, providing an important clue regarding the effect of SIRT3 in POCD in aged mice.

Considering the negative regulation of SIRT3 in hippocampal neuroinflammation, we speculated that anesthesia/surgery induced a mitochondrial oxidative stress response in a SIRT3-dependent manner, resulting in microglia activation and hippocampal neuroinflammation and thus reducing synaptic plasticity in the CA1 region and postoperative cognition in aged mice.

There are several limitations in our study. First, in the current work, we only focused on cognitive function in the early postoperative period; therefore, the effect on long-term outcomes of brain cognitive function should be investigated in further studies. Second, different kinds of anesthesia and surgical treatment procedures may also influence SIRT3 expression, the inflammatory response, and cognitive performance in mice. However, in the current project, we did not include a separate anesthesia control group. We reasoned that because a patient who undergoes an operation will experience all these procedures, all of these factors may serve to imitate the whole treatment plan in clinical management. Other perioperative factors related to SIRT3 expression and neuroinflammation in the hippocampus of aged mice should be determined in further studies. Third, the results of fear conditioning and open field tests in mice do not directly correlate with cognitive function in humans. Fourth, although there are three types of SOD enzymes (CuZn-SOD [SOD1], Mn-SOD [SOD2], and EC-SOD [SOD3]), we measured the total SOD enzymatic activity to study oxidative stress in the hippocampus of aged mice under anesthesia/surgery. We found that Giuseppa Mudo et al. also employed total SOD enzymatic activity measurements in a rat model of AD [65]. Whether the SOD enzyme activity dysfunction induced by anesthesia/surgery is dependent on SOD enzyme type remains to be further investigated. Finally, we could not fully exclude other potential molecules that might be involved in SIRT3-mediated POCD in aged mice.

## Conclusion

In conclusion, the current study indicated that SIRT3 may attenuate postoperative cognitive decline by preventing the anesthesia/surgery-induced neuroinflammatory response in the hippocampal CA1 region of aged mice. SIRT3 may be a potential therapeutic and diagnostic target for the management of POCD in aged patients.

## Supplementary Information

**Additional file 1.** Supplementary information.

## Data Availability

The data supporting the findings of this study are presented within the manuscript.

## Abbreviations

SIRT3: Sirtuin 3
POCD: Postoperative cognitive dysfunction
SOD: Superoxide dismutase
MDA: Malondialdehyde
Iba1: Ionized calcium binding adapter molecule 1
TNF-α: Tumor necrosis factor-alpha
IL-1β: Interleukin (IL)-1β
IL-6: Interleukin (IL)-6
BBB: Blood–brain barrier
AAV: Adeno-associated virus
OFT: Open field test
FCT: Fear conditioning test
BCA: Bicinchoninic acid
ELISA: Enzyme-linked immune-sorbent assay
OD: Optical density
fEPSPs: Field excitatory postsynaptic potentials
TBS: Theta burst stimulation
PNDs: Perioperative neurocognitive disorders
PD: Parkinson’s disease
AD: Alzheimer’s disease
LTP: Long-term potentiation
