# Supplementary material for: Sirtuin 3 protects against anesthesia/surgery-induced cognitive decline in aged mice by suppressing hippocampal neuroinflammation
Source: J Neuroinflammation. 2021 Feb 4;18:41. doi: 10.1186/s12974-021-02089-z (PMC7863360; doi:10.1186/s12974-021-02089-z)

SIRT3

SIRT3

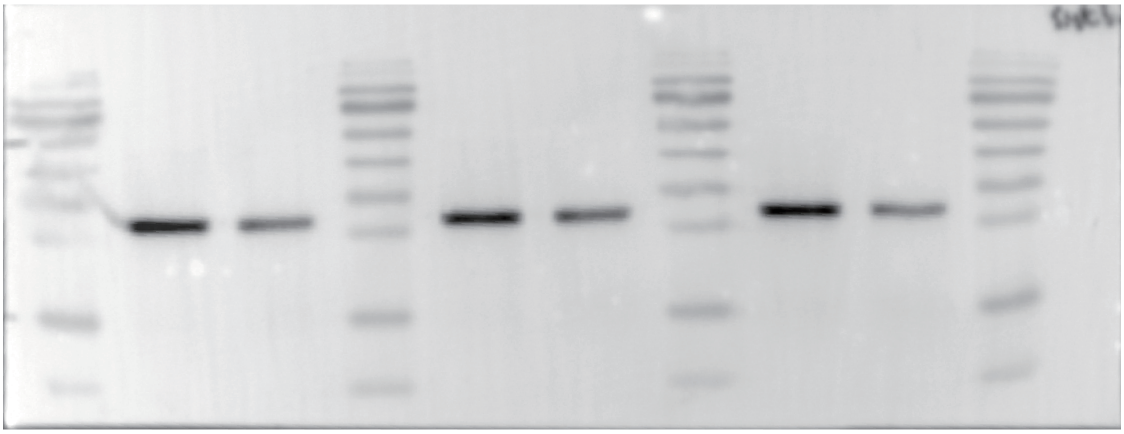

28 kDa

marker | C | A/S | marker | C | A/S | marker | C | A/S | marker

SIRT1

SIRT1

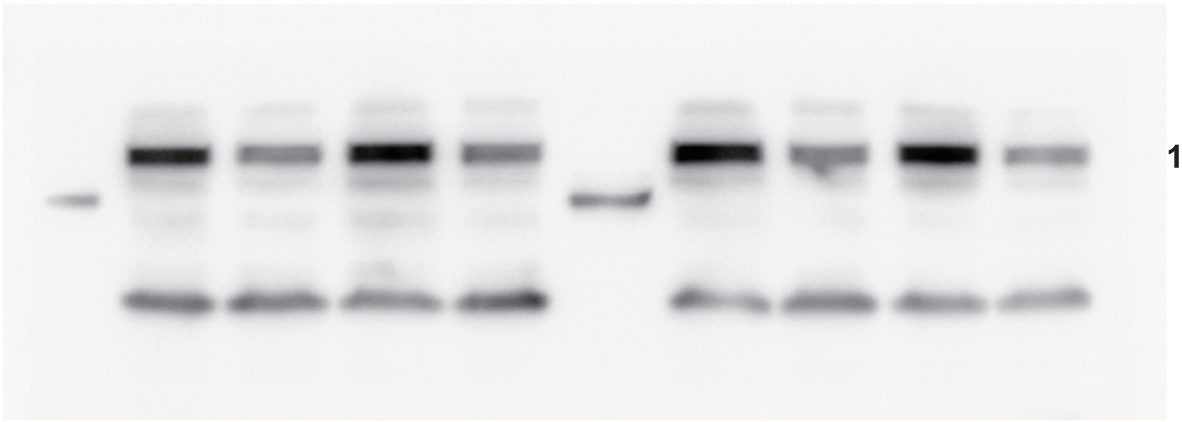

110 kDa

marker | C+VEH | A/S+VEH | C+SIRT3 | A/S+SIRT3 | marker | C+VEH | A/S+VEH | C+SIRT3 | A/S+SIRT3

Iba1

Iba1

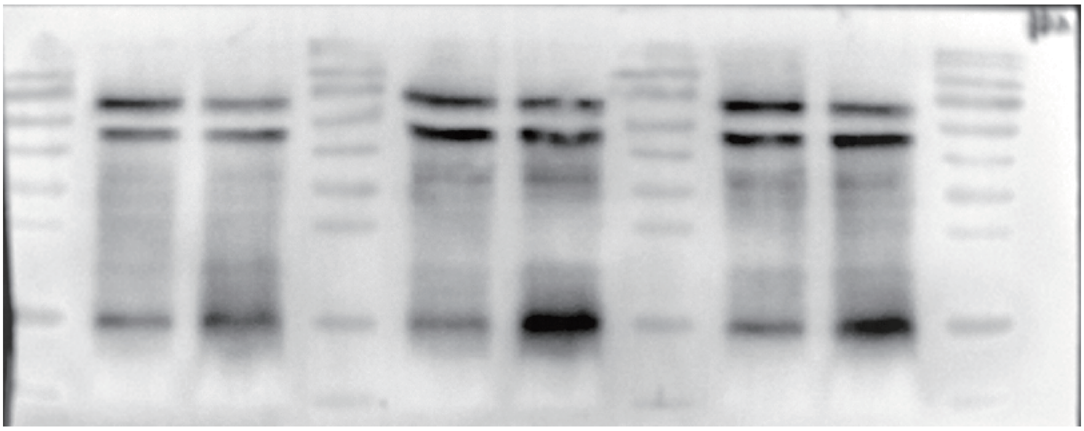

17 kDa

marker | C | A/S | marker | C | A/S | marker | C | A/S | marker

Fig 3A SIRT3 Day1

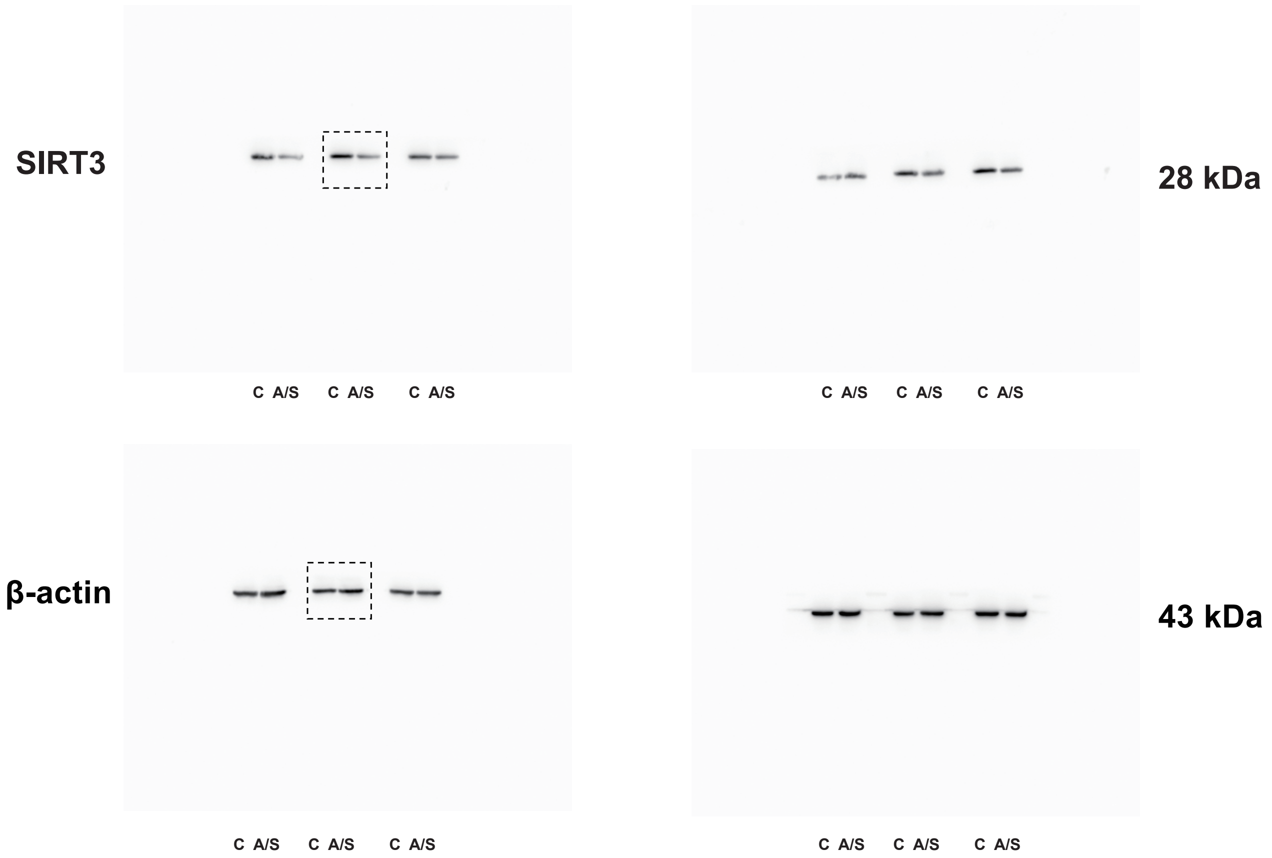

Fig 3B SIRT3 Day3

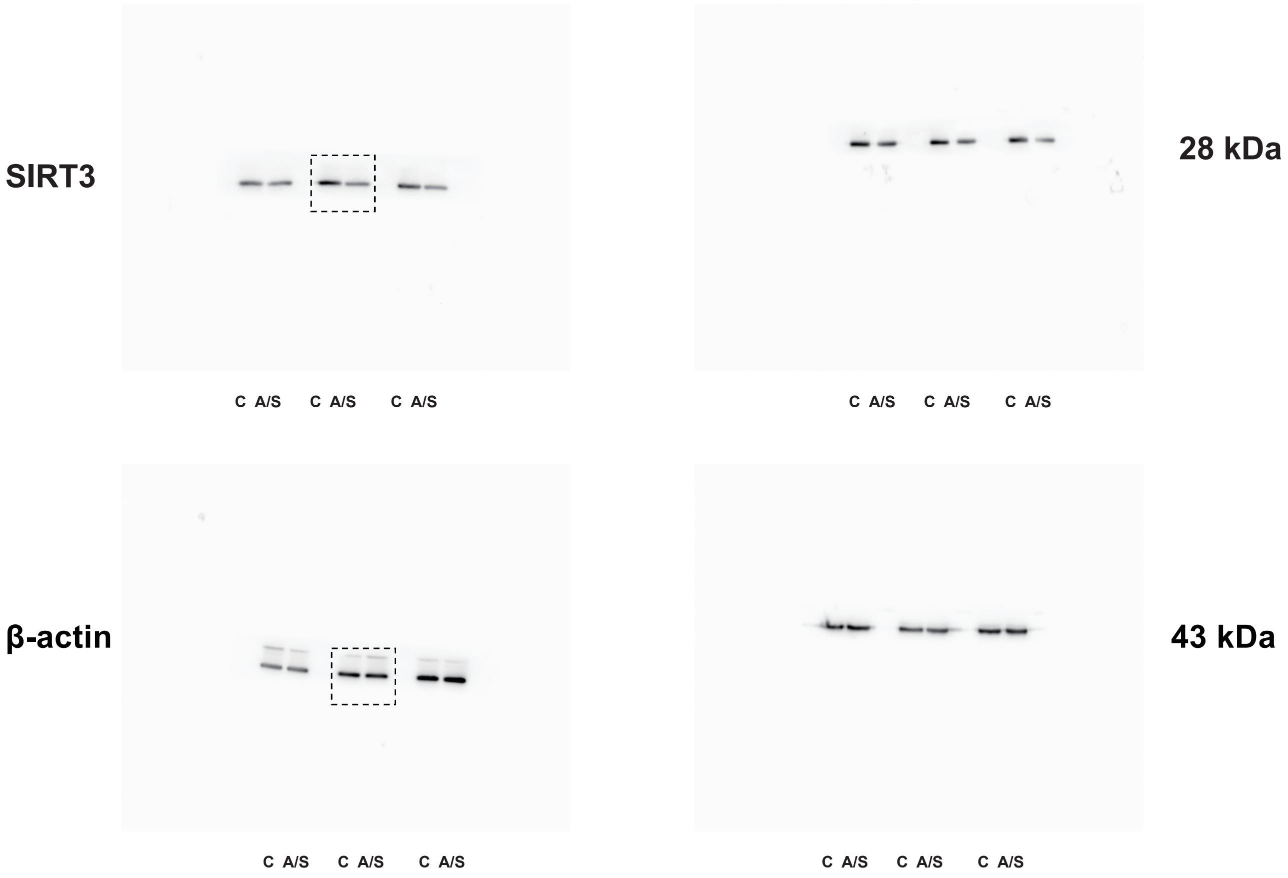

Fig 3C SIRT3 Day7

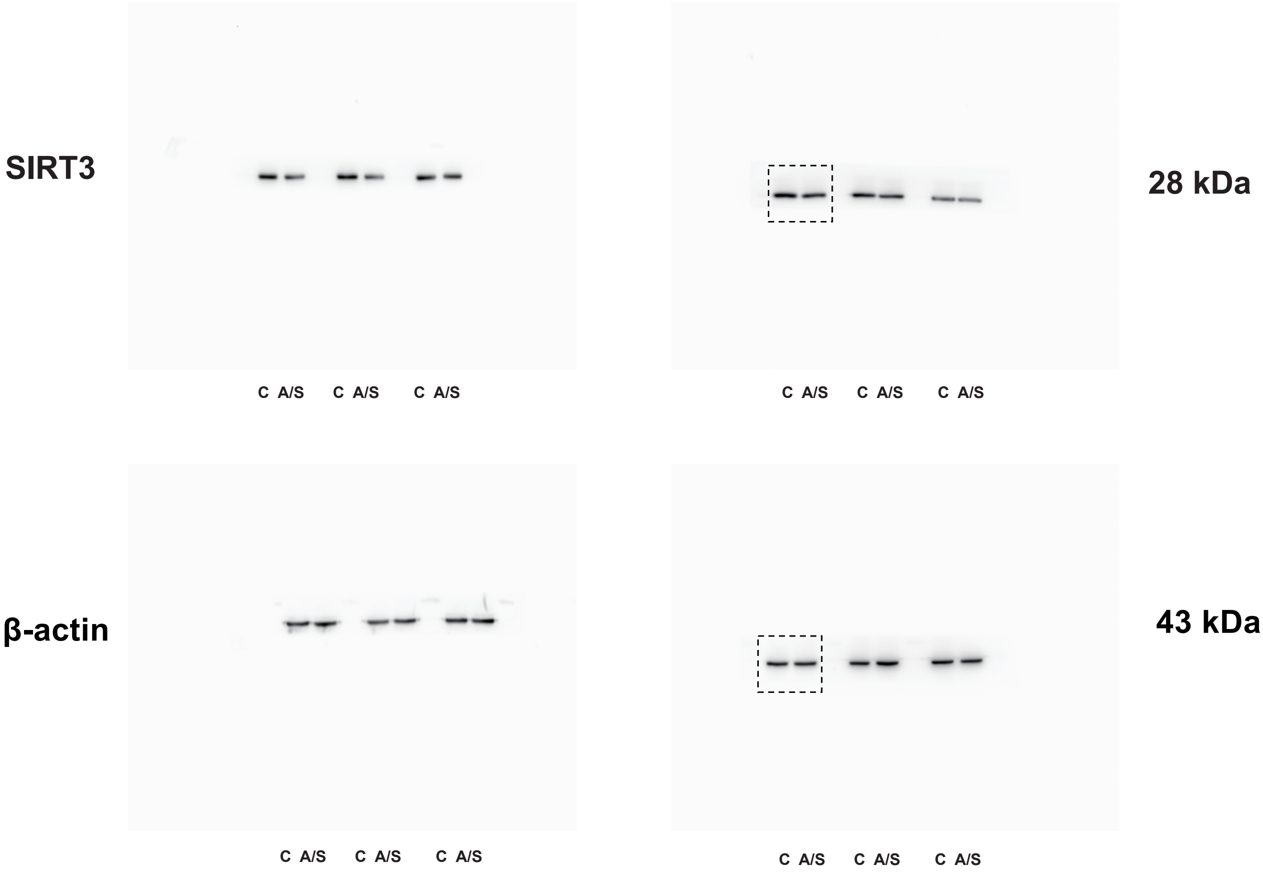

Fig 4C Iba1

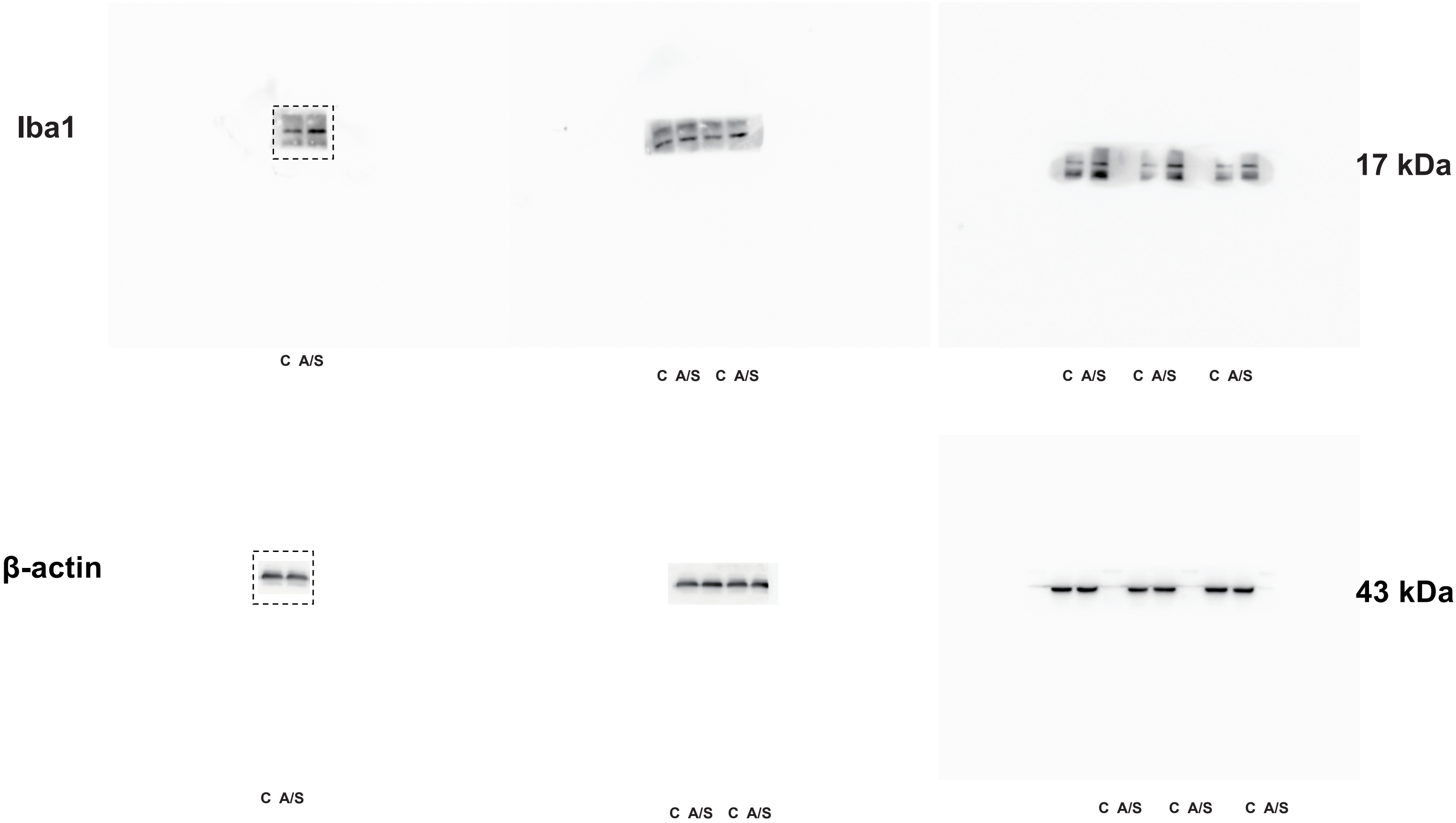

Fig 6C SIRT3 Day1

1 C + VEH
2 A/S + VEH
3 C + SIRT3
4 A/S + SIRT3

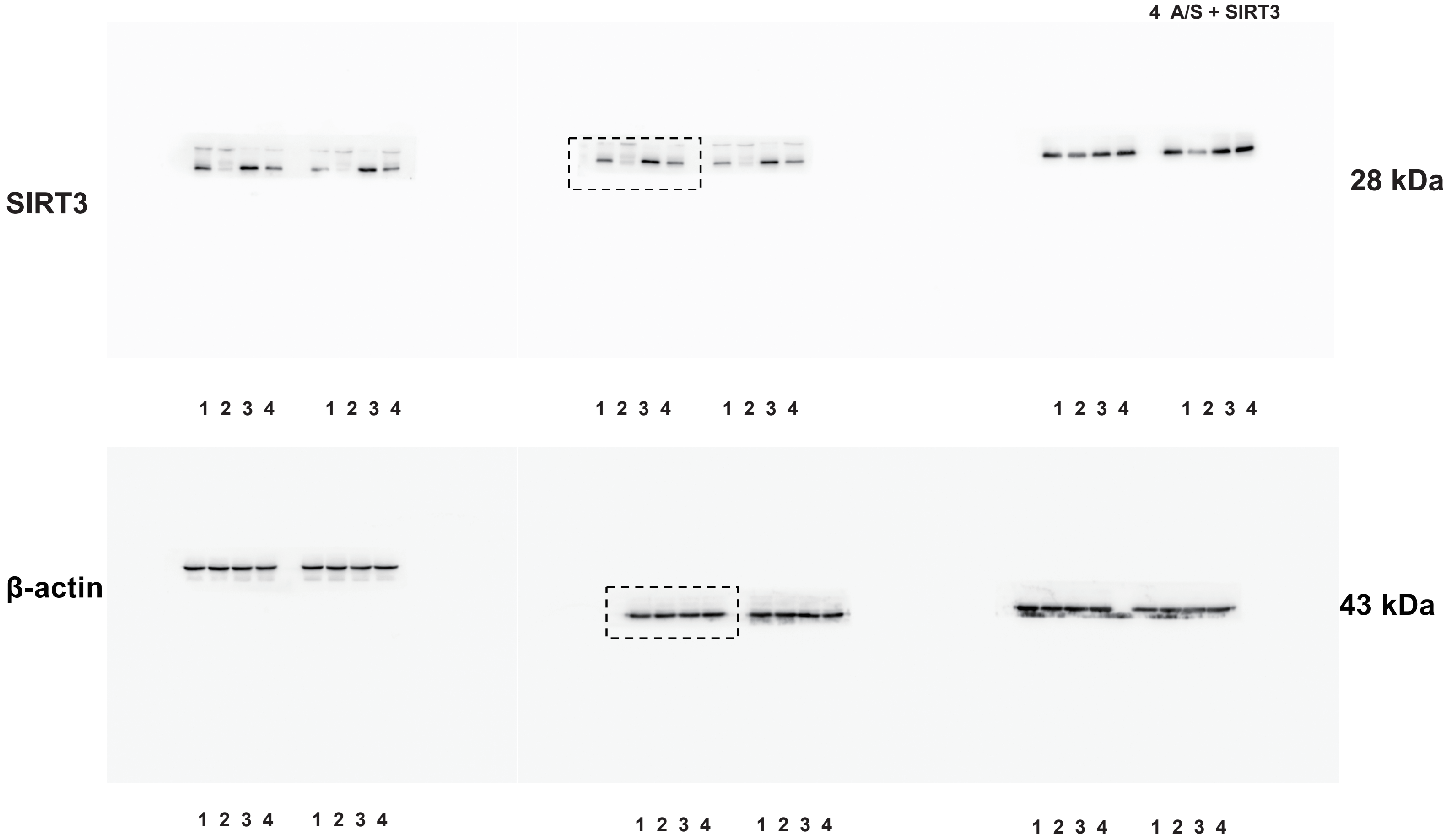

Fig 6D SIRT3 Day3

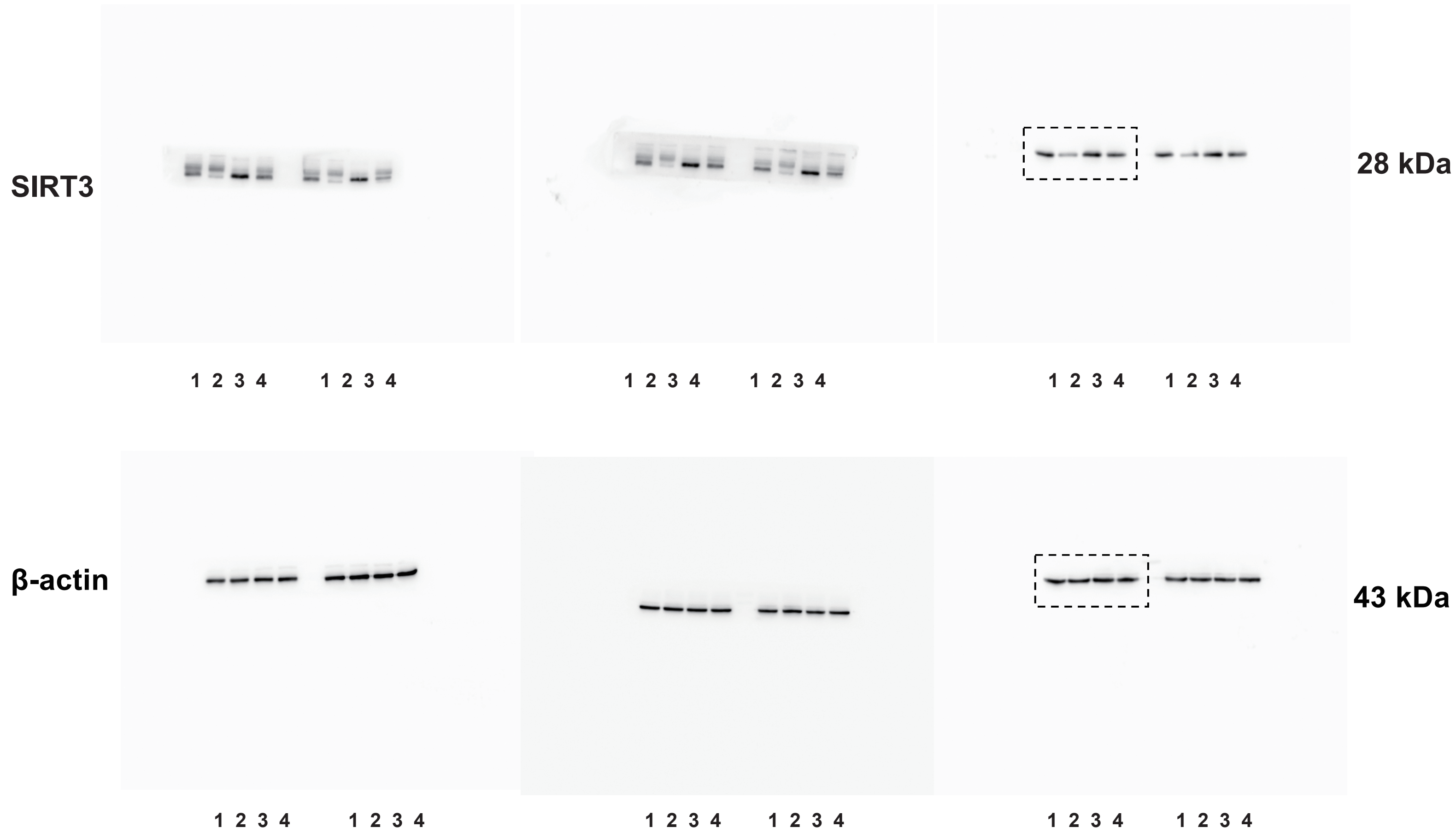

Fig 6E SIRT3 Day7

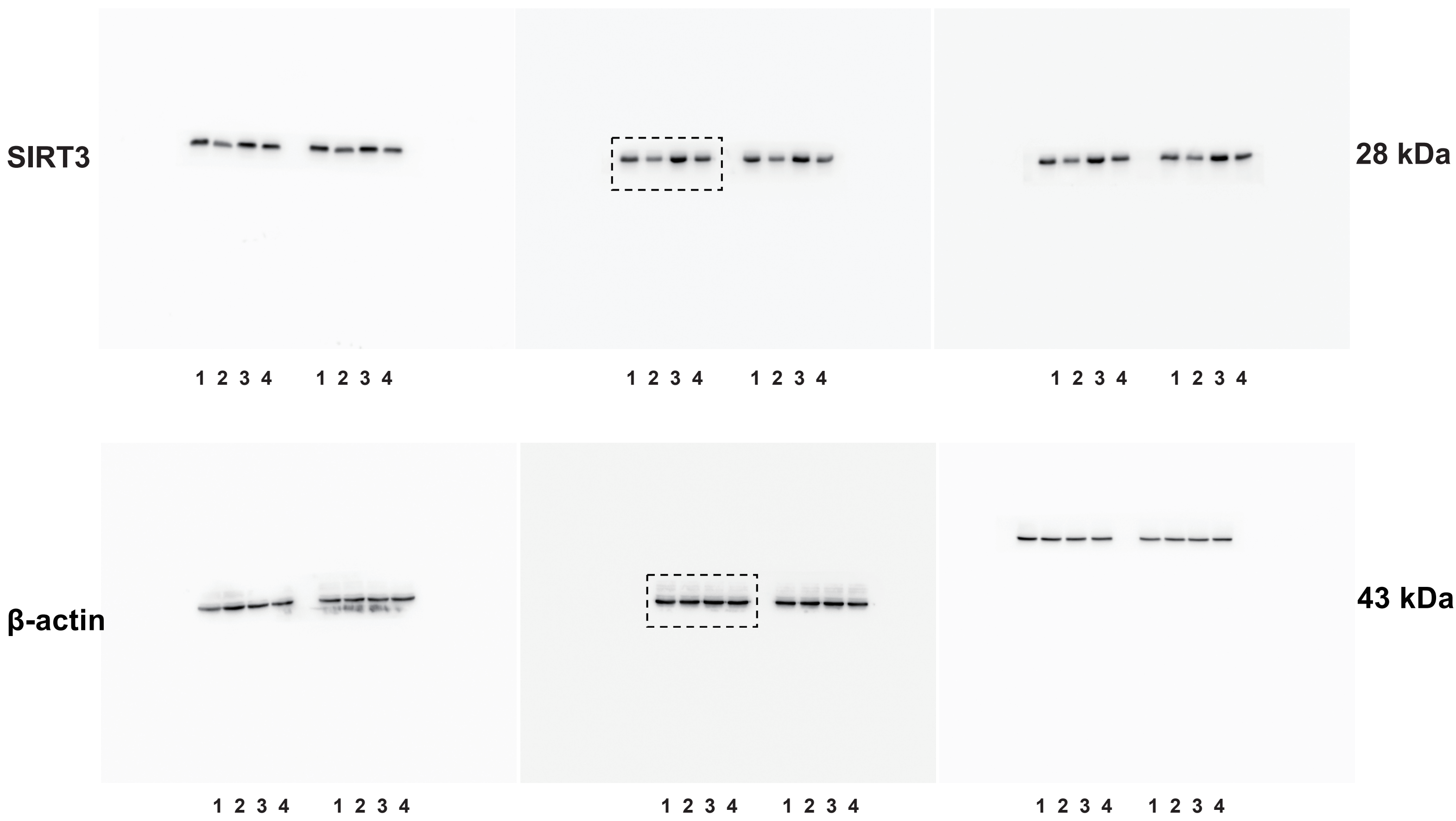

Fig 7C Iba1

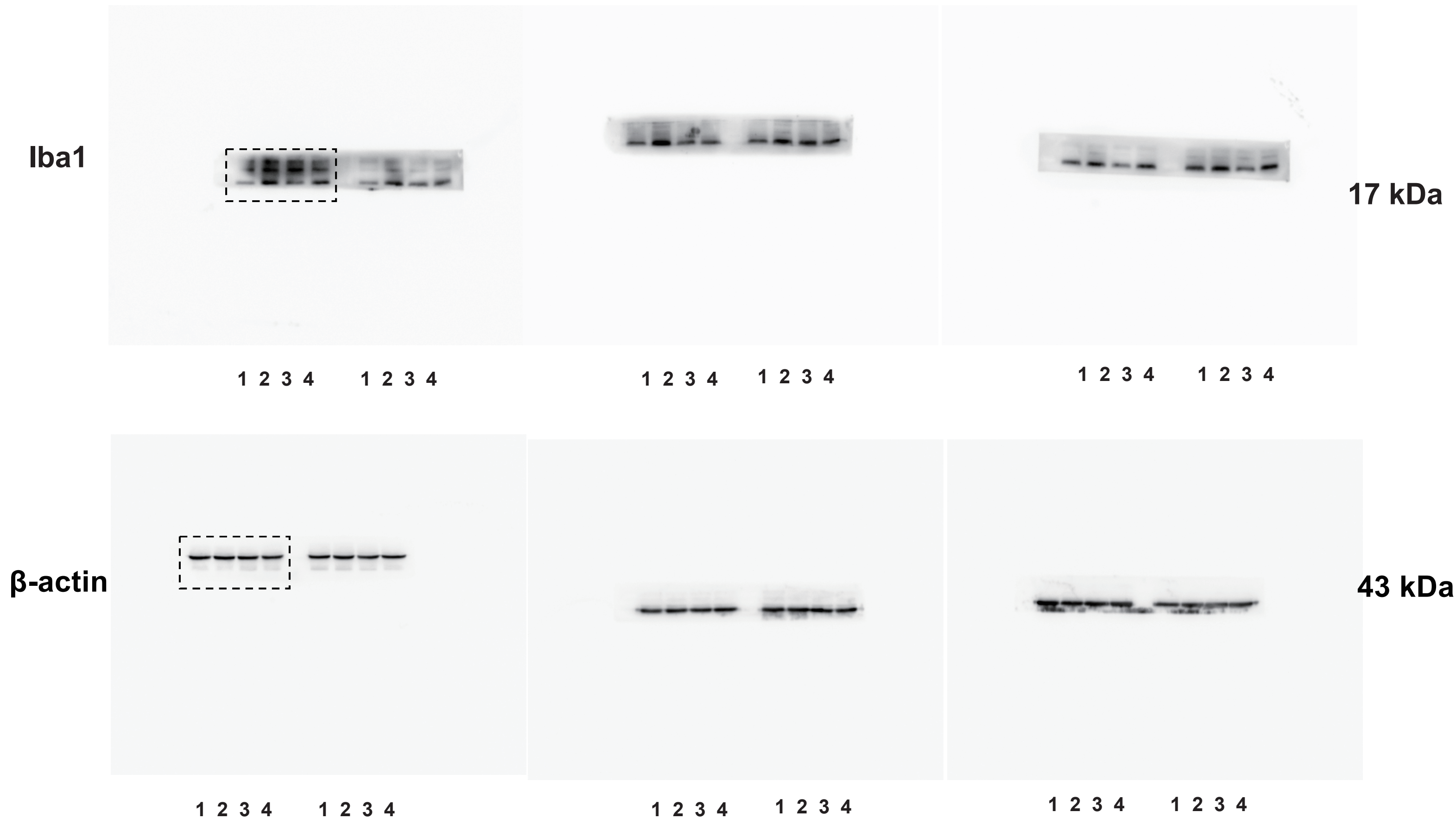

Fig 6K SIRT1 Day1

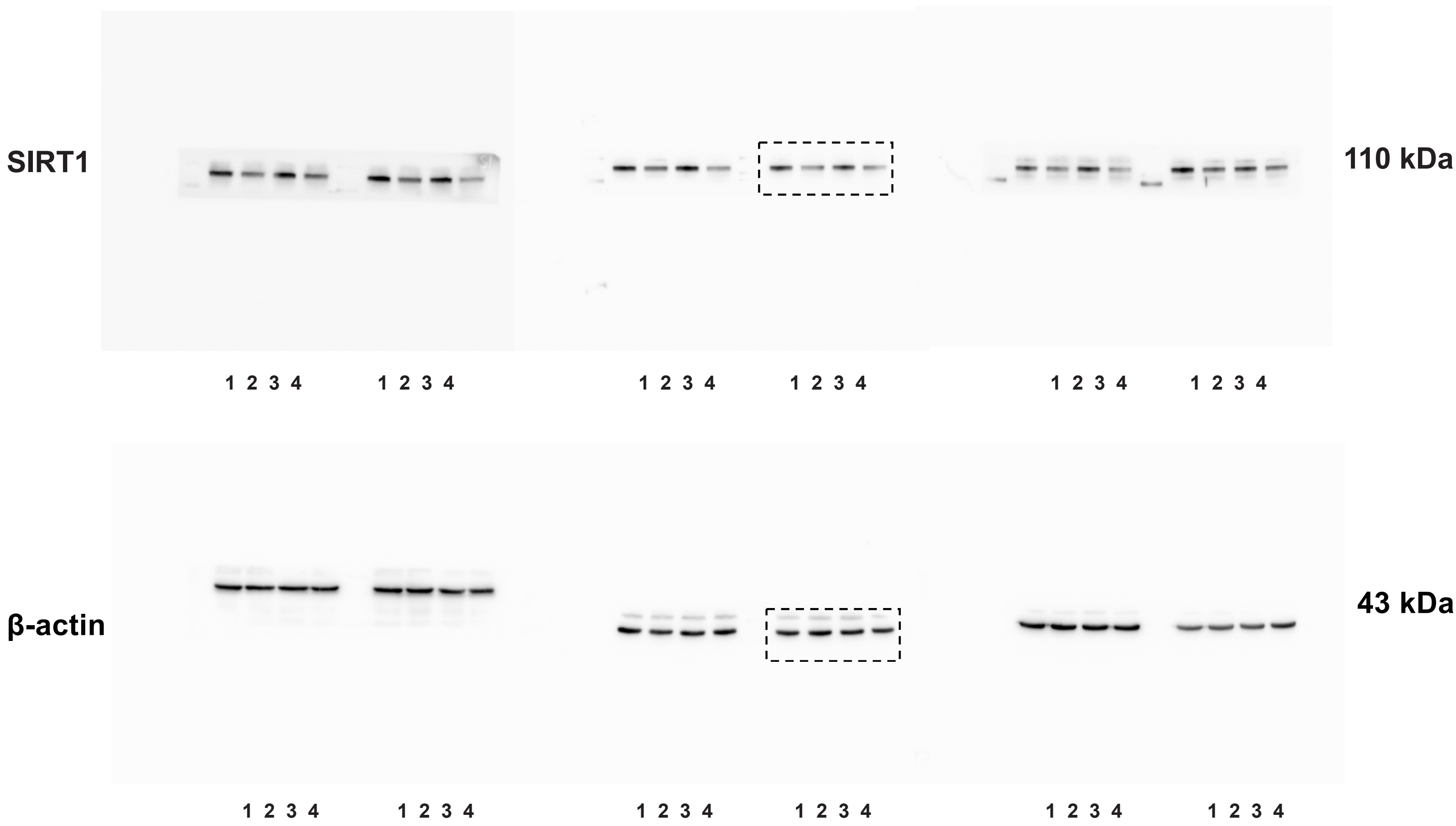

Fig 6L SIRT1 Day3

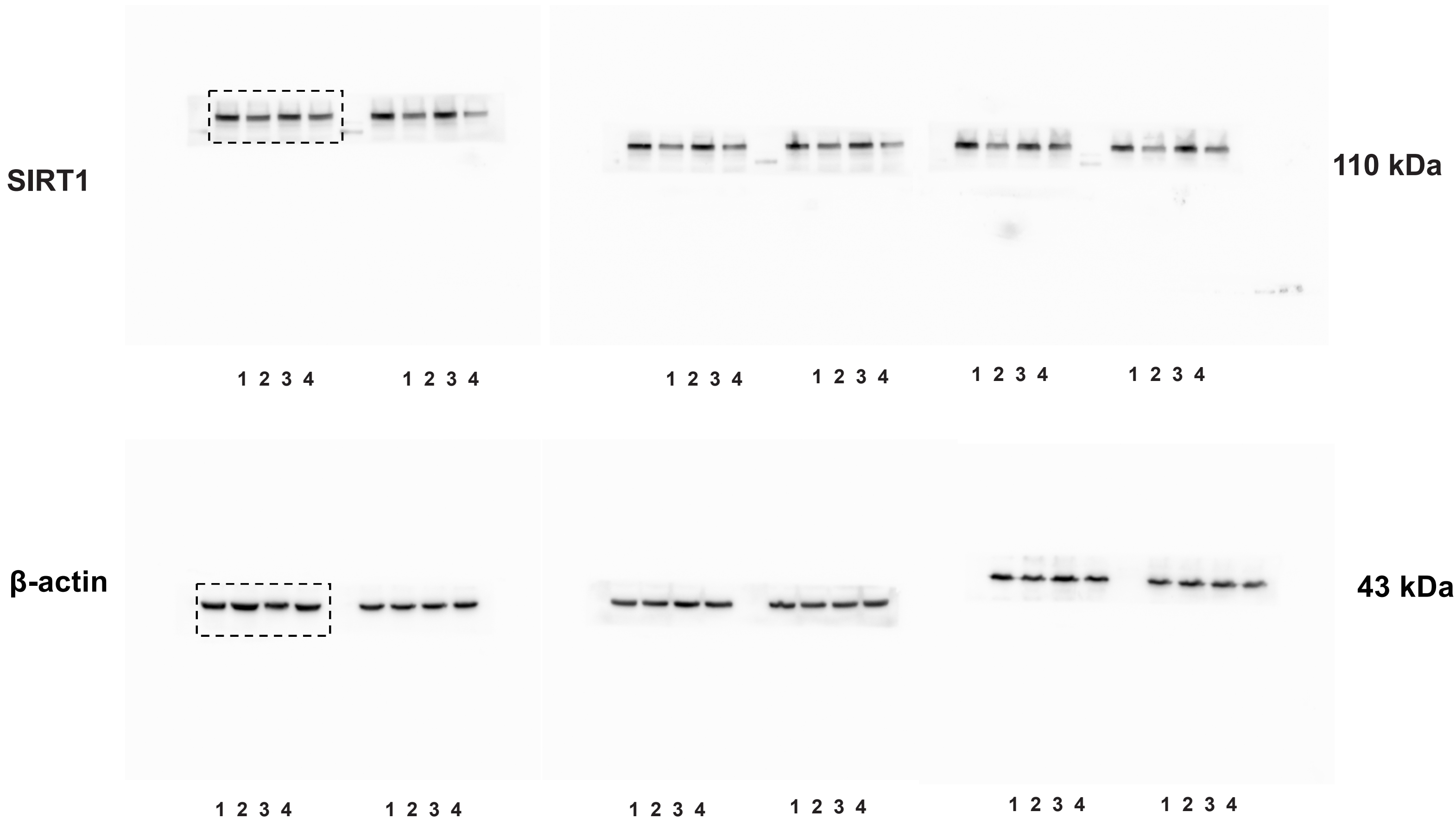

Fig 6M SIRT1 Day7

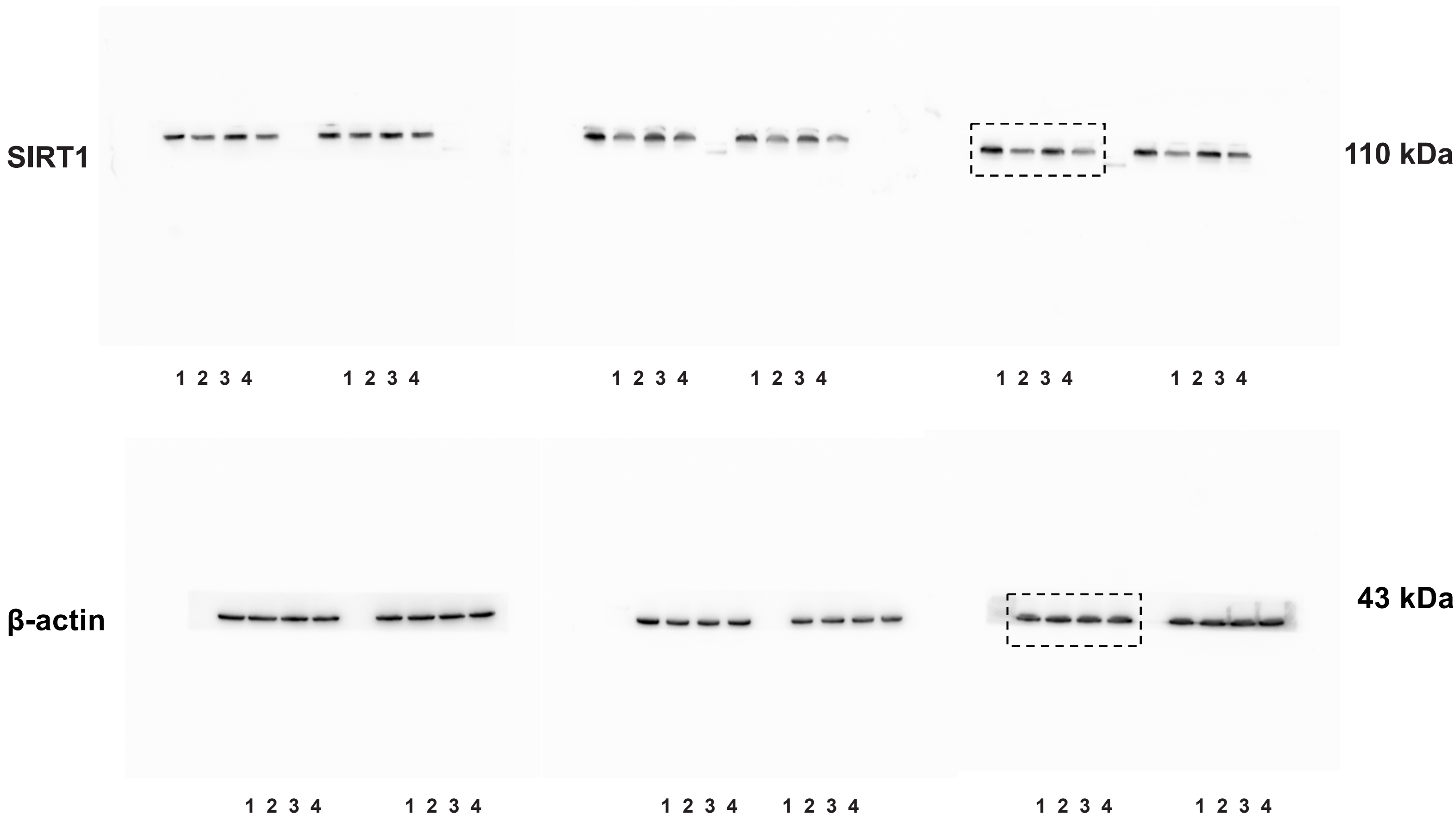

Supplement: Supplementary file 1 — Additional file 1. Supplementary information. [file 12974_2021_2089_MOESM1_ESM.pdf]
